# *In Situ* Cocrystallization via Spray Drying with Polymer as a Strategy to Prevent Cocrystal Dissociation

**DOI:** 10.1021/acs.molpharmaceut.3c00564

**Published:** 2023-08-18

**Authors:** ShiZhe Shao, Michael W. Stocker, Salvatore Zarrella, Timothy M. Korter, Abhishek Singh, Anne Marie Healy

**Affiliations:** † School of Pharmacy and Pharmaceutical Sciences, Trinity College Dublin, Dublin D02 PN40, Ireland; ‡ SSPC, the Science Foundation Ireland Research Centre for Pharmaceuticals, Trinity College Dublin, Dublin D02 PN40, Ireland; § Department of Chemistry, Syracuse University, 1-014 Center for Science and Technology, Syracuse, New York 13244, United States; ∥ Janssen Pharmaceutica NV, Beerse 2340, Belgium; ⊥ School of Chemical and Bioprocess Engineering, University College Dublin, Dublin D04 V1W8, Ireland

**Keywords:** Spray drying, Cocrystal, Crystalline solid dispersion, Polymer, Dissociation

## Abstract

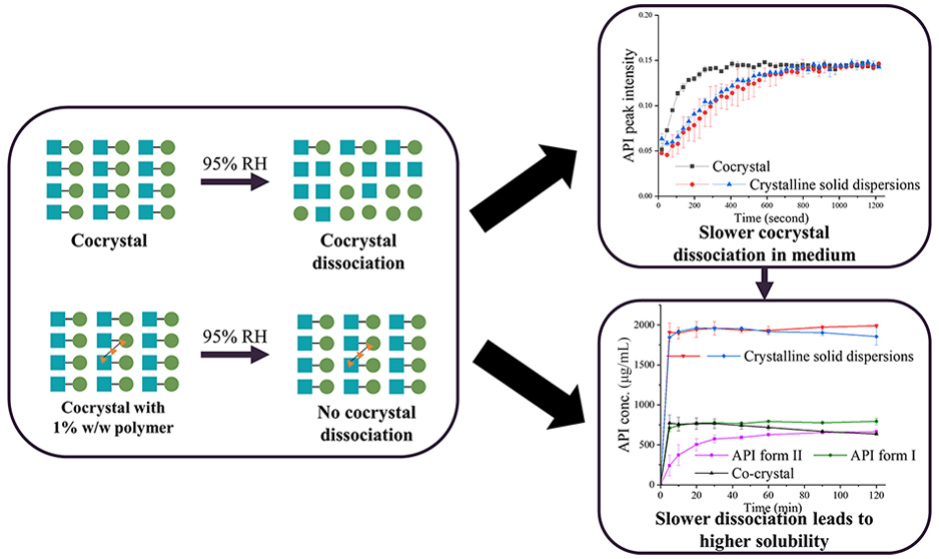

The aim of the present study was to investigate how different polymers affect the dissociation of cocrystals prepared by co-spray-drying active pharmaceutical ingredient (API), coformer, and polymer. Diclofenac acid–l-proline cocrystal (DPCC) was selected in this study as a model cocrystal due to its previously reported poor physical stability in a high-humidity environment. Polymers investigated include polyvinylpyrrolidone (PVP), poly(1-vinylpyrrolidone-*co*-vinyl acetate) (PVPVA), hydroxypropyl methyl cellulose, hydroxypropylmethylcellulose acetate succinate, ethyl cellulose, and Eudragit L-100. Terahertz Raman spectroscopy (THz Raman) and powder X-ray diffraction (PXRD) were used to monitor the cocrystal dissociation rate in a high-humidity environment. A Raman probe was used *in situ* to monitor the extent of the dissociation of DPCC and DPCC in crystalline solid dispersions (CSDs) with polymer when exposed to pH 6.8 phosphate buffer and water. The solubility of DPCC and solid dispersions of DPCC in pH 6.8 phosphate buffer and water was also measured. The dissociation of DPCC was water-mediated, and more than 60% of DPCC dissociated in 18 h at 40 °C and 95% RH. Interestingly, the physical stability of the cocrystal was effectively improved by producing CSDs with polymers. The inclusion of just 1 wt % polymer in a CSD with DPCC protected the cocrystal from dissociation over 18 h under the same conditions. Furthermore, the CSD with PVPVA was still partially stable, and the CSD with PVP was stable (undissociated) after 7 days. The superior stability of DPCC in CSDs with PVP and PVPVA was also demonstrated when systems were exposed to water or pH 6.8 phosphate buffer and resulted in higher dynamic solubility of the CSDs compared to DPCC alone. The improvement in physical stability of the cocrystal in CSDs was thought to be due to an efficient mixing between polymer and cocrystal at the molecular level provided by spray drying and *in situ* gelling of polymer. It is hypothesized that polymer chains could undergo gelling *in situ* and form a physical barrier, preventing cocrystal interaction with water, which contributes to slowing down the water-mediated dissociation.

## Introduction

Advanced drug discovery approaches in use today have resulted in the structures of active pharmaceutical ingredients (APIs) becoming much more complex, which has increased the possibility of them presenting poor physicochemical and biopharmaceutical properties. Around half of the APIs on the market and 90% of APIs in early phase development studies have poor solubility, which can lead to unfavorable dissolution performance and a lack of efficacy.^1^ Engineering APIs into different solid state forms, such as anhydrates, solvates, hydrates, salts, and cocrystals has demonstrated potential for enhancing the properties of the APIs.^2^ Among these solid forms, cocrystals are a well-studied solid form, which are defined by the US Food and Drug Administration (FDA) as “crystalline materials composed of two or more different molecules, typically API and cocrystal formers (“coformers”), in the same crystal lattice”.^3^ With the involvement of the coformer molecule, the properties of cocrystals are unlikely to be similar to the API itself.^4,5^ For instance, seven carbamazepine cocrystals reported by Good and Rodríguez-Hornedo showed an aqueous solubility from 2 to 152 times higher than the stable carbamazepine dihydrate form.^6^ An indomethacin–saccharin cocrystal also showed a higher intrinsic dissolution rate at pH 1.2 and 7.4 and higher bioavailability in dogs compared to the parent compound.^7^

Despite the improvement in the biopharmaceutical properties of cocrystals over APIs, there are concerns about the physical stability of cocrystals. As cocrystals generally contain two or more components in the same crystal lattice, the dissociation of cocrystals into API and coformer molecules or the transformation from cocrystals to other solid forms may occur upon storage,^8^ especially when there is a large difference in aqueous solubility between API and coformer.^9^ Recent studies have found that certain excipients, when simply mixed with a cocrystal, could induce the dissociation of a “stable” cocrystal (with no excipients added) during the course of a stability study.^10−12^ Duggirala et al. concluded that the effect of excipient-induced dissociation might be related to the different pH_eq_ (surface acidity) and hygroscopicity of the excipients. Concerns over the stability of cocrystals in a liquid environment have also been raised, as the dissociation of cocrystal before complete dissolution may hinder the advantages presented by the cocrystal of high solubility and high dissolution rate.^13,14^ Strategies to prevent or retard cocrystal dissociation still lack study and need an in-depth understanding, which will be beneficial for the development of pharmaceutical cocrystal formulations on an industrial scale.

The approaches to synthesizing cocrystals are currently categorized into liquid-based methods and mechanochemical methods (non-liquid-based methods). The former utilizes organic solvent to assist the cocrystallization of cocrystals from API and coformer(s) and includes solution evaporation and slurry methods.^15,16^ The latter involves mechanochemical neat grinding and liquid-assisted grinding,^17,18^ which promote the chemical reaction between solids induced by mechanical energy^19^ and cocrystallization of the cocrystals from a coamorphous system.^20^ Most studies have paid more attention to small-scale production and synthesis of the cocrystals; however, several scalable techniques, such as spray drying,^21,22^ fluidized bed granulation/coating,^23,24^ and hot melt extrusion,^25^ have been explored with respect to the possibility of formulating cocrystal solid dispersions (cocrystal molecularly dispersed with an excipient) by one-step cocrystallization from API, coformer, and suitable excipient.

Spray drying is well-known for the formation of amorphous solid dispersions (ASDs), as spray drying is a process that generates micronized powder by the fast evaporation of small, atomized droplets. Previous studies have proven that spray drying can generate pure cocrystals from incongruent saturating conditions.^21,26^ One-step preparation of cocrystal formulations by spray drying has not been widely studied. Some studies have indicated that spray-dried cocrystal formulations could further improve the physicochemical and biopharmaceutical properties of cocrystals.^22,27,28^ How spray drying conditions can affect the production of cocrystal formulations has also been investigated.^29^ Overall, spray drying as a scalable technique has shown potential for the preparation of cocrystal formulations in a one-step process and is worthy of further investigation.

The aim of this study was to investigate how different polymers affect cocrystal production and stability when co-spray-dried with API and coformer and if crystalline solid dispersions (CSDs) can be prepared with different polymers and different weight fractions of polymer, whereby the cocrystal remains in the crystalline form when coprocessed with polymer. Furthermore, how different polymers affect the cocrystal dissociation and whether cocrystal–polymer solid mixtures generated by simply blending spray-dried cocrystal and polymer would dissociate at a similar rate as co-spray-dried systems (CSDs) are also the focus of this study.

Diclofenac acid–l-proline cocrystal (DPCC) was selected for this study. Diclofenac acid (DA) is a nonsteroidal anti-inflammatory drug and is commonly used in its potassium and sodium salt forms because of the poor solubility and dissolution properties of the free acid form.^30,31^ A zwitterionic diclofenac acid–l-proline cocrystal (DPCC) was previously reported by Nugrahani et al. as showing improved aqueous solubility relative to diclofenac but poor physical stability above 75% relative humidity (RH) at 30 °C.^32^

In the present study, DPCC and DPCC CSDs were prepared by spray drying from ethanol. Polymers investigated were polyvinylpyrrolidone K25 (PVP), poly(1-vinylpyrrolidone-*co*-vinyl acetate) (PVPVA), hydroxypropyl methyl cellulose (HPMC), hydroxypropylmethylcellulose acetate succinate (HAS), ethyl cellulose (EC), and Eudragit L-100 (EUD). Powders were characterized by powder X-ray diffraction (PXRD), thermogravimetric analysis (TGA), Fourier transform infrared spectroscopy (FTIR), terahertz Raman spectroscopy (THz Raman), and fingerprint region Raman spectroscopy with a PhAT probe (PhAT Raman). The dynamic solubility of cocrystal in water and pH 6.8 phosphate buffer was also measured and cocrystal alone compared to cocrystal in CSDs.

## Materials and Methods

### 1.1 Materials

Diclofenac acid (DA) (DA form II)^33^ and anhydrous l-proline (PROAH) were purchased from Glentham Life Science (Corsham, UK). DA form I was prepared following the method previously reported by King et al. (and involved the rapid addition of water to a saturated DMSO solution at room temperature).^33^ PVP (Kollidon K25, 28 000–34 000 g/mol) and PVPVA (Kollidon VA 64, 45 000–70 000 g/mol) were provided by BASF (Ludwigshafen, Germany). HPMC (27–30% methoxy content and 7–12% hydroxypropoxy content, 40–60 cP, around 22 000 g/mol) was purchased from Alfa Aesar (Ward Hill, Massachusetts, United States). HPMCAS-HF (HAS) (Aquasolve, 75 100 g/mol) was purchased from Shin-Etsu Chemical Co., Ltd. (Tokyo, Japan). Eudragit L-100 (EUD) (approximately 125 000 g/mol) was purchased from Evonik Industries (Essen, Germany). Ethylcellulose (EC) (Ethocel Standard 4 Premium, around 19 200 g/mol) was a gift from Dow Chemical Company (Bomlitz, Germany). Sodium chloride was purchased from Fluorochem (Hadfield, UK). 85% phosphoric acid solution, sodium phosphate monohydrate, and potassium phosphate monobasic were purchased from Sigma-Aldrich (Wicklow, Ireland). Disodium hydrogen phosphate was purchased from Merck Millipore (Darmstadt, Germany). Absolute alcohol, HPLC grade methanol, and HPLC grade water were purchased from Fisher Scientific Ltd. (Loughborough, UK). The purified water was deionized and prepared using a Milli-Q integral system and a Millipore Elix advantage system (Merck, Rahway, New Jersey, United States).

### 1.2 Preparation Methods

#### 1.2.1 Spray Drying

2 g of DA form II and PRO was dissolved at a stoichiometric molar ratio of 1:1 (1.440 g of DA and 0.560 g of PRO), either solely or codissolved with 20, 60, and 100 mg of polymer, respectively (Table 1), in 40 mL of ethanol at 65 °C in a 50 mL DURAN closed bottle (DWK LIFE SCIENCE, Mainz, Germany). The bottle with the suspension was kept in a water bath at 65 °C, heated by a hot plate. The suspension in the bottle was stirred at 600 rpm. Once the suspension became clear, the solution was kept at 65 °C and spray-dried using a Büchi Mini Spray Dryer B-290 (Büchi, Flawil, Switzerland) with a high-performance cyclone (Büchi, Flawil, Switzerland) running in open mode. A two-fluid nozzle with a 1.5 mm nozzle cap and a 0.7 mm nozzle tip was used. Solutions were spray-dried at an inlet temperature of 78 °C, an aspirator rate of 100% (35 m^3^/h), an atomization rate of 667 L/h, and a liquid feed rate of 10% (3 mL/min). The outlet temperature was in the range of 48–54 °C.

**Table 1:** Formulations Prepared by Spray Drying

Abbreviation	Polymer used	Polymer amount
SDDPCC	/	N/A
SDPVP1	PVP	20 mg (1% mass of DA and PRO)
SDPVP3	PVP	60 mg (3% mass of DA and PRO)
SDPVP5	PVP	100 mg (5% mass of DA and PRO)
SDPVPVA1	PVPVA	20 mg (1% mass of DA and PRO)
SDPVPVA3	PVPVA	60 mg (3% mass of DA and PRO)
SDPVPVA5	PVPVA	100 mg (5% mass of DA and PRO)
SDHPMC1	HPMC	20 mg (1% mass of DA and PRO)
SDHPMC3	HPMC	60 mg (3% mass of DA and PRO)
SDHPMC5	HPMC	100 mg (5% mass of DA and PRO)
SDHAS1	HAS	20 mg (1% mass of DA and PRO)
SDHAS3	HAS	60 mg (3% mass of DA and PRO)
SDHAS5	HAS	100 mg (5% mass of DA and PRO)
SDEC1	EC	20 mg (1% mass of DA and PRO)
SDEC3	EC	60 mg (3% mass of DA and PRO)
SDEC5	EC	100 mg (5% mass of DA and PRO)
SDEUD1	EUD	20 mg (1% mass of DA and PRO)
SDEUD3	EUD	60 mg (3% mass of DA and PRO)
SDEUD5	EUD	100 mg (5% mass of DA and PRO)

#### 1.2.2 Physical Mixes (PMs) of Cocrystal and Polymers

A 100 mg portion of spray-dried DPCC (SDDPCC) was physically mixed with either 1 or 10 mg of each polymer (i.e., 1 or 10% by mass of SDDPCC) (Table 2) in a capped type IB neutral glass vial (outer diameter of 20 mm, height of 42 mm) (Fisher Scientific Ltd., Loughborough, UK) using a TURBULA type T2 C (Glen Creston Ltd., Stanmore, UK) blender at 67 rpm for 5 min. The same blender was also used for mixing 100 mg of DA and PROAH in a stoichiometric molar ratio of 1:1 (72.0 mg of DA and 28.0 mg of PROAH) (Table 2, PMDAPRO).

**Table 2:** Physical Mixes (PMs) Prepared by Blending

Abbreviation	Polymer used	Polymer amount
PMDAPRO	/	0
PMPVP1	PVP	1 mg (1% mass of SDDPCC)
PMPVP10	PVP	10 mg (10% mass of SDDPCC)
PMPVPVA1	PVPVA	1 mg (1% mass of SDDPCC)
PMPVPVA10	PVPVA	10 mg (10% mass of SDDPCC)
PMHPMC1	HPMC	1 mg (1% mass of SDDPCC)
PMHAS1	HAS	1 mg (1% mass of SDDPCC)
PMEC1	EC	1 mg (1% mass of SDDPCC)
PMEUD1	EUD	1 mg (1% mass of SDDPCC)

### 1.3 Characterization Approaches

#### 1.3.1 Powder X-ray Diffraction (PXRD)

A Miniflex II Rigaku Diffractometer (Rigaku, Japan) with Ni-filtered Cu Kα radiation (1.54 Å) was used for the PXRD analysis. The tube voltage and tube current were 30 kV and 15 mA, respectively. Powder was evened out on a zero-background silicon sample holder. The diffractogram was collected between 5 and 40° 2 theta (2θ). For qualitative purposes (*n* = 2), a step size of 0.05° and a stepping time of 1 s were used. For quantitative phase analysis (QPA) by Rietveld refinement, a step size of 0.02° and a stepping time of 1 s were used.

#### 1.3.2 Thermogravimetric Analysis (TGA)

A TGA Q50 instrument (TA Instruments, Elstree, UK) was used for residual moisture content (RMC) and residual solvent content (RSC) measurements. 5–10 mg of sample was added to an open aluminum pan for TGA analysis. The sample was heated to 180 °C with a 10 °C/min ramping rate. Universal analysis software (TA Instruments, Elstree, UK) was used for analyzing the RMC and RSC, which were defined as the weight percentage loss from ambient temperature up to 140 °C. The analysis was repeated twice.

#### 1.3.3 Raman Spectroscopy

##### 1.3.3.1 Fingerprint Spectra Collected Using the PhAT Probe

A RamanRxn instrument (Endress, Reinach, Switzerland) equipped with a PhAT probe (Endress, Reinach, Switzerland) with a spot diameter of 6 mm at the focal position was used for the Raman spectroscopy analysis. The samples were exposed to the laser at a power of 400 mW and a wavelength of 785 nm. The Raman spectra were collected over six scans and 2 s of exposure time. Spectra were acquired over the fingerprint region from 150 to 1850 cm^–1^.

##### 1.3.3.2 Low-Frequency Raman Spectra Collected Using the THz-PROBE

A RIO 50 Plus probe (HORC, Michigan, United States) attached to an Ondax THz-Raman probe module (Coherent, Santa Clara, California, United States) was used for collecting Raman spectra. Samples were exposed to the laser with a power of 100 mW and a wavelength of 808 nm. Six scans and 10 s of exposure time were used. The Raman spectra were collected in the Raman region with spectral resolution of 4–6 cm^–1^ from −1200 to 2900 cm^–1^. Spectragryph software version 1.2 (developed by Dr. Friedrich Menges, Oberstdorf, Germany) was used for Raman data analysis.^34^ The spectra were processed by the advanced baseline function at a coarseness of 15%. The *x*-offset for each spectrum was corrected by the Rayleigh line. Spectra were normalized based on the peak of highest intensity for comparison purposes.

Estimation of the cocrystal (%) content in the mixture was accomplished using a peak height ratio model, built using THz Raman spectra. Physical mixtures composed of SDDPCC and DA form II were prepared and blended using a TURBULA type T2 C (Glen Creston Ltd., Stanmore, UK) for 5 min at 67 rpm before use. The calibration curve (*R*^2^ > 0.99), based on 22 experimental points (11 different concentrations, prepared in duplicate), was built, with the proportion of crystalline (undissociated) DPCC (cocrystal%) in the DPCC and DA form II mixture on the *x*-axis and the relative intensity, calculated from eq 1 below, on the *y*-axis.
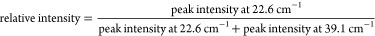
1

##### 1.3.3.3 Low-Frequency Raman Spectra Collected Using the THz-BENCH

An Ondax XLF-CLM THz-Raman spectrometer (Coherent, Santa Clara, California, United States) was used to take both 295 K (room temperature) and 78 K (liquid nitrogen) Raman spectral data for DA, PRO, and DPCC. The system is based on laser excitation centered at λ = 784.7 nm and detection using an Andor Shamrock DR-750 spectrograph (Oxford Instruments, Abingdon, UK) equipped with a cooled iDus 416 CCD camera (Oxford Instruments, Abingdon, UK). Finely ground crystalline powder was placed in a Lake Shore ST-100 vacuum cryostat (Lake Shore Cryotronics, Westerville, Ohio, United States) using a cuvette system with glass windows that enabled free-space laser access and scattering collection. Final Raman spectra are composed of 225 acquisitions of 3 s exposure times, with a spectral range of 5 to 300 cm^–1^ and a spectral resolution of 0.6 cm^–1^. Atmospheric interference from N_2_ and O_2_ rotational Raman scattering was identified and subtracted from the presented spectra by using Spectragryph software (version 1.2).

#### 1.3.4 Fourier Transform Infrared Spectroscopy (FTIR)

A PerkinElmer Spectrum 1 FT-IR Spectrometer (PerkinElmer, Waltham, United States) equipped with an UATR and a ZnSe crystal accessory was used for FTIR analyses. 30 scans were performed in the range of 650–4000 cm^–1^ with a resolution of 4 cm^–1^.

#### 1.3.5 Particle Size Analysis

A Mastersizer 3000 (Malvern Instruments Ltd., Worcestershire, UK) equipped with an Aero S dry powder disperser unit was used for particle size analysis. The analysis was undertaken by using an air pressure of 2.0 bar and a feed rate of 50%. The d10, d50, and d90 particle size parameters are reported, representing the diameters corresponding to 10, 50, and 90%, respectively, of the cumulative undersize volume distribution. Measurements were performed in triplicate and analyzed using Mastersizer 3000 software (Version 5.61).

#### 1.3.6 Scanning Electron Microscopy (SEM)

A Zeiss Supra variable pressure field emission SEM instrument (Zeiss, Oberkochen, Germany) was used to study particle morphology. Powder was attached to the carbon adhesive discs, mounted on an aluminum stub, and sputter coated with gold before analysis. Powder was analyzed at an electron high tension voltage of 1.5–2 kV.

### 1.4 Pharmaceutical Properties Investigation

#### 1.4.1 Dynamic Solubility (DS) Study

The dynamic solubility study was performed in jacketed vessels (60 mL capacity) with a water bath attached (Lauda, Lauda-Königshofen, Germany). Excess amounts (around 2.5–3 times the solubility of each solid) were added to 20 mL of HPLC grade water or pH 6.8 phosphate buffer (0.2 M)^35^ at 37 °C in a jacketed vessel. A 20 × 6 mm magnet was utilized to provide and maintain a ∼600 rpm stirring rate for the suspension in each jacketed vessel. 1 mL of suspension was taken out from the jacketed beaker at 5, 10, 20, 30, 45, 60, 90, and 120 min and filtered through a 0.45 μm PTFE filter (Fisherbrand, Waltham, MA, United States) followed by appropriate dilution. All the diluted liquid samples were then analyzed by HPLC using the method detailed in Section 1.4.2. These experiments were performed in triplicate.

The suspension was filtered after completion of the study. The filtered solid samples from the triplicate experiments were collected, dried in an ambient environment, gently mixed and ground in a mortar, and then analyzed by PXRD for the QPA study (Sections 1.3.1 and 1.5.2). The filtered liquid samples from the triplicate experiments were mixed, and pH was measured by an Orion Versa Star Pro pH meter (Thermo Fisher Scientific, Waltham, MA, United States).

#### 1.4.2 High-Performance Liquid Chromatography (HPLC)

An Alliance HPLC (Waters, Milford, Massachusetts, United States) with a Waters 2695 separation module system (Waters, Milford, Massachusetts, United States) and a Waters 2996 photodiode array detector (Waters, Milford, Massachusetts, United States) was used for DA concentration quantification. The HPLC method followed that detailed in the United States Pharmacopeia (USP) monograph.^36^ A HyPURITY C18 HPLC column (Thermo Fisher Scientific, Waltham, MA, United States) with a 150 × 4.6 mm inner length and diameter and 5 μm particle size was used. The mobile phase was composed of a solution (pH 2.5 ± 0.2) of 0.01 M phosphoric acid and 0.01 M monobasic sodium phosphate and a solution of HPLC grade methanol (70:30, v/v). The injection volume was 20 μL, and the flow rate was 1 mL/min. The wavelength detector used a wavelength of 254 nm. The column was kept at ambient temperature.

100 μg/mL DA stock solution was prepared in a solution of methanol and water (70:30, v/v). Various diluted DA solutions of 50, 25, 12.5, 6.25, 3.125, and 1.5625 μg/mL were prepared by adding water as a diluent. A calibration curve (*R*^2^ > 0.999) generated from the eluted DA peak area at 6.2 min and the concentration of DA was used for DA concentration determination in solution.

#### 1.4.3 Monitoring Cocrystal Stability When Exposed to Liquid Medium

Around 3 mg of powder was pressed onto the die (0.5 cm diameter) of the zero-background silicon XRD sample holder. 60 μL of pH 6.8 phosphate buffer or 40 μL of HPLC grade water was added onto the surface of the compact. The Raman signal of the compacted powder was collected by the PhAT probe immediately after the liquid was added to the surface. The Raman signal was collected every 30 s for 20 min. The study was performed in duplicate.

Spectragryph software version 1.2 was used for data analysis. Spectra between 150 and 300 cm^–1^ were cut off before the spectra were processed by the advanced baseline function at a coarseness of 15%. The spectra were then normalized based on the peak intensity at 1578 cm^–1^.

The powders from the duplicate analyses were added to a mortar, gently ground, and mixed with a pestle. The powder was then tested by PXRD for the QPA study.

#### 1.4.4 Solid-State Stability Study

100 mg of powder was added into a 7 mL uncapped type IB neutral glass vial (outer diameter of 20 mm, height of 42 mm) (Fisher Scientific Ltd., Loughborough, UK). The powder was evened out in the glass vials, which were then placed in a sealed glass chamber (size approximately 19 × 19 × 10 cm).

The 95% relative humidity (RH) of the sealed chamber was established by saturated potassium sulfate solution to achieve 95 ± 5% RH. The chamber was placed in an oven (Gallenkamp, Loughborough, UK) at 40 ± 2 °C. A sensor (Sensirion AG, Stafa, Switzerland) was placed in the sealed chamber to monitor the temperature and RH of the chamber.

Samples from the stability study were analyzed by PXRD for qualitative analysis and THz-PROBE Raman for quantitative analysis after 2, 6, 12, 18 h, and 7 days storage and TGA for RMC determination after 18 h and 7 days of storage. Discrete, fresh samples were prepared for each time point. The accelerated stability study was performed in duplicate.

#### 1.4.5 Imaging of Powder/Gel in Bulk

The images of powder/gel in the vials (outer diameter of 20 mm, height of 42 mm) (Fisher Scientific Ltd., Loughborough, UK) were captured by a USB microscope 9 MP (Conrad Electronic, Hirschau, Germany) using the eScope software program (OiTez, Hongkong, China).

### 1.5 Modeling, Properties Prediction, and Calculation

#### 1.5.1 Simulated PXRD Patterns

Simulated PXRD patterns were calculated from single crystal data files with Mercury software version 3.10.3 (Cambridge Crystallographic Data Centre, UK). The reference crystal structures were downloaded from the Cambridge Crystallographic Data Centre (CCDC). The reference structures used in this study include DPCC with a Refcode of RETNEM01,^37^ DA form I with a Refcode of SIKLIH09,^33^ DA form II with a Refcode of SIKLIH10,^33^ DA form III with a Refcode of SIKLIH04,^38^l-proline anhydrate (PROAH) with a Refcode of PROLIN02,^39^ and l-proline monohydrate (PROHY) with a Refcode of RUWGEV.^40^

#### 1.5.2 Rietveld Refinement for QPA Estimation

X’Pert HighScore Plus version 3.0 (Malvern Panalytical, Malvern, UK) was used for Rietveld refinement.^41^ The Rietveld refinement method used followed that reported by McCusker et al.^42^ The background was determined by manually adding base points. A semiautomatic Rietveld refinement mode was utilized. Scale factor, zero shift, and unit cell parameters were first refined for peak position correction. The pseudo-Voigt function was used for peak profile refinement. Peak shape parameters, including U, V, and W, and peak shape factor 1 were then secondarily refined. The preferred orientation was the last option to be refined. The quality of Rietveld refinement was evaluated by goodness-of-fit (GOF) and Rwp (weighted-profile residual values) but mainly by viewing the difference between the observed and calculated pattern, as recommended by Toby.^43^

#### 1.5.3 Theoretical Computational Methods

The CRYSTAL17 software package^44^ was used to complete solid-state density functional (ss-DFT) simulations of DA form II, PROAH, and DPCC. These quantum mechanical calculations utilize periodic boundary conditions to account for the three-dimensional environment of the crystalline solids. All starting structures were obtained from the Cambridge Structural Database^45^ and the Refcodes for DA, PRO, and DPCC were SIKLIH10,^33^ PROLIN05,^46^ and RETNEM01,^37^ respectively. All calculations were performed within the published space group symmetries of *C*2*/c* for DA, P212121 for PRO, and *P*21 for DPCC.

For all ss-DFT calculations, the Perdew–Burke–Ernzerhof (PBE) density functional was used in conjunction with the VTZp basis set.^47,48^ The calculations were augmented by the addition of Grimme’s London dispersion correction (D3) utilizing the Becke–Johnson damping correction (BJ)^49−51^ and three-body repulsion Axilrod–Teller–Muto repulsion contributions (program keyword: “ABC”).^52,53^ A pruned integration grid of 99 radial points and 1454 angular points (program keyword “XXLGRID”) was also implemented. The k-point counts in the irreducible Brillouin zones of the crystals were 100 for DA, 125 for PRO, and 170 for DPCC. The overlap-based truncation criteria for the bielectronic integrals were set to 10^–10^, 10^–10^, 10^–10^, 10^–10^, 10^–10^, and 10^–20^.

Full structural optimizations were performed with an energy convergence threshold of Δ*E* < 10^–8^ E_h_. Subsequent vibrational frequency calculations were performed with a stricter energy convergence of Δ*E* < 10^–10^ E_h_. The vibrational analyses were based on displacements of the atoms of the crystallographic asymmetric unit cell, each displaced twice along the Cartesian axes for determination of the numerical derivatives of the Hessian matrix via the central difference formula. Raman intensities were calculated using the coupled-perturbed Hartree–Fock/Kohn–Sham (CPHFKS) method.^54−56^ To aid comparison with experimental measurements, the calculated frequency positions and Raman intensities of the normal modes of vibration were convolved with empirical Lorentzian line shapes with a full-width-at-half-maximum (fwhm) of 2 cm^–1^ for direct comparison of the spectra.

## Results and Discussion

### 1.6 Characterization of Spray-Dried Solid Dispersions

#### 1.6.1 PXRD Analysis

**Figure 1:**
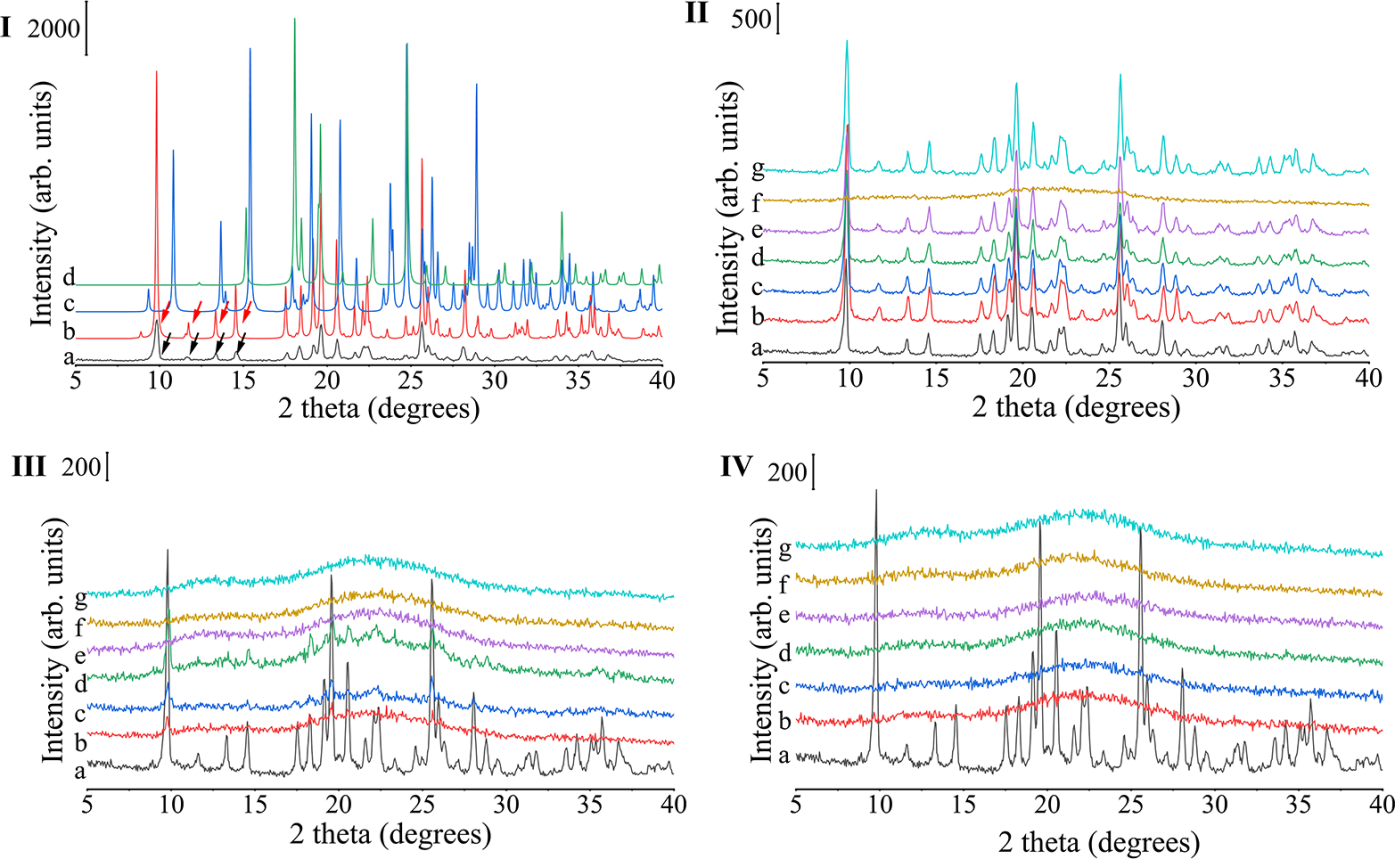
(I) PXRD patterns of (a) SDDPCC and simulated PXRD patterns of (b) SDDPCC, (c) DA form II, and (d) PROAH. (II) PXRD patterns of (a) SDDPCC, (b) SDPVP1, (c) SDPVPVA1, (d) SDHPMC1, (e) SDHAS1, (f) SDEC1, and (g) SDEUD1. (III) PXRD patterns of (a) SDDPCC, (b) SDPVP3, (c) SDPVPVA3, (d) SDHPMC3, (e) SDHAS3, (f) SDEC3, (g) SDEUD3. (IV) PXRD patterns of (a) SDDPCC, (b) SDPVP5, (c) SDPVPVA5, (d) SDHPMC5, (e) SDHAS5, (f) SDEC5, (g) SDEUD5.

For the production of DPCC, several methods have been reported previously, including neat grinding,^57^ liquid-assisted grinding,^32,37^ solvent evaporation,^32,37^ use of microwave,^58^ and antisolvent crystallization.^57^ Compared to traditional solvent evaporation, spray drying is a technique that produces a powder by evaporating the solvent droplets at a higher temperature for a much shorter period of time. In the present study, the diffractogram of SDDPCC presented DPCC characteristic peaks at 9.8, 11.7, and 14.5° 2θ (as shown by the black and red arrows in Figure 1-I) and did not show additional characteristic peaks of DA and PRO, indicating that spray drying could generate physically pure DPCC (Figure 1-I).

How different types and amounts of polymers affect the formation of the DPCC via the spray drying process was also investigated. Interestingly, the inclusion of polymers at levels of only 1–5 wt % by total mass of DA and PRO resulted in different spray-dried solid forms. PXRD patterns of spray-dried products containing 1 wt.% polymer (Figure 1-II) were the same as the diffractogram of SDDPCC, which indicated the formation of the DPCC was not affected by the inclusion of 1 wt.% polymer when the polymer is PVP, PVPVA, HPMC, HAS, or EUD, and CSDs can be produced. Nevertheless, the solid dispersions prepared by spray drying with EC were different from the solid dispersions prepared with other polymers. The diffractograms (Figure 1-II–IV) of SDEC1, SDEC3, and SDEC5 showed broad halo patterns, indicating that as little as 1 wt % of EC could amorphize DA and PRO resulting in ASDs. With 5 wt % polymer, all solid dispersions showed an amorphous halo, indicating ASDs were formed (Figure 1-IV). With 3% polymers, SDHAS3, SDEUD3, and SDEC3 were also ASDs (Figure 1-III). Very small crystalline Bragg peaks at 8.9 and 14.5° 2θ, representing crystalline DPCC, can be seen in the diffractograms of SDHPMC3, SDPVP3, and SDPVPVA3, indicating that 3 wt % of HPMC, PVP, and PVPVA resulted in semicrystalline solid dispersions (sCSD). Thus, all cocrystal solid dispersions prepared with polymer could be classified into different categories based on their PXRD-identified solid state, as shown in Table 3.

**Table 3:** Classification of Cocrystal Solid Dispersions Based on PXRD Diffractograms

Classification of solid dispersions	Formulations
Crystalline solid dispersions (CSDs)	SDPVP1, SDPVPA1, SDHPMC1, SDHAS1, SDEUD1
Semicrystalline solid dispersions (sCSDs)	SDPVP3, SDPVPVA3, SDHPMC3
Amorphous solid dispersions (ASDs)	SDEC1, SDHAS3, SDEC3, SDEUD3, SDPVP5, SDPVPVA5, SDHPMC5, SDHAS5, SDEC5, SDEUD5

#### 1.6.2 Raman Analysis in the Low-Frequency Region

When compared to conventional Raman spectroscopy, THz Raman spectroscopy can not only provide molecular structure information from the fingerprint region but also collect crystalline structure information from the THz region.^59^ The fingerprint region spectra collected using the THz-PROBE had a much lower Raman intensity compared to those attained using the PhAT probe (Figure S3), so the analysis in the fingerprint region utilized only the Raman spectra collected by the PhAT probe (Figure S2, Table S3), as discussed in the Supporting Information.

**Figure 2:**
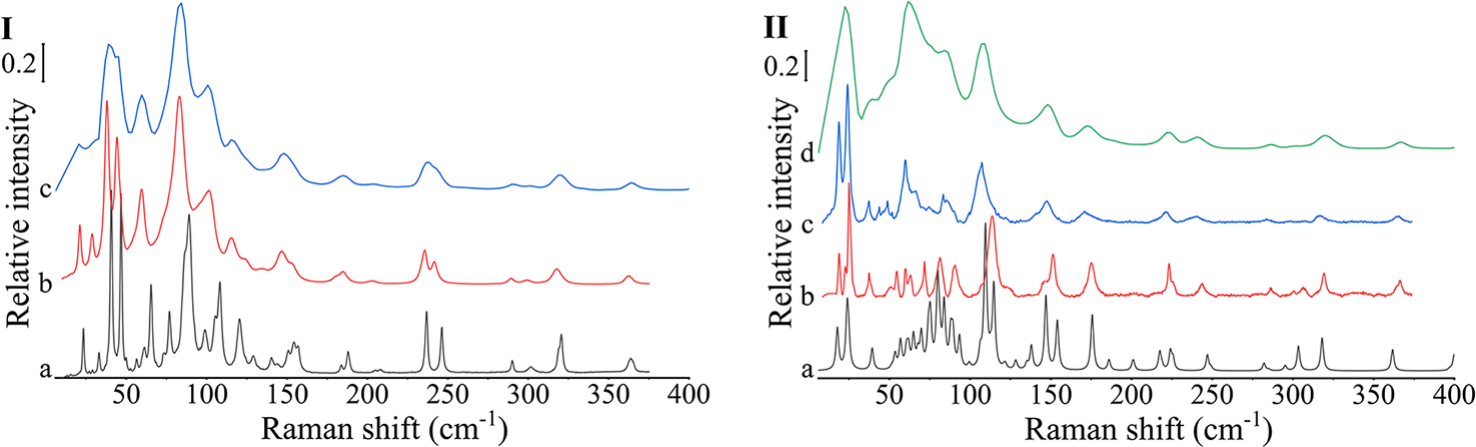
(I) Raman spectra below 400 cm^–1^ of (a) DA form II at 78 K collected using the THz-BENCH, (b) DA form II at 295 K collected using the THz-BENCH, and (c) DA form II at 295 K collected using the THz-PROBE. (II) Raman spectra below 400 cm^–1^ of (a) DPCC simulated by ss-DFT, (b) DPCC at 78 K collected using the THz-BENCH, (c) DPCC at 295 K collected using the THz-BENCH, and (d) DPCC at 295 K collected using the THz-PROBE

Both THz-PROBE and THz-BENCH were utilized for the analysis of DA form II and DPCC (Figure 2). The spectra of DA form II and SDDPCC using the THz-PROBE were in good agreement with the spectra collected using the THz-BENCH, but the spectra collected by THz-PROBE were a “smoothened” version of the THz-BENCH spectra, as the THz-PROBE setup has a 2 cm^–1^ step width, which is much wider than that of the THz-BENCH, which has a 0.2 cm^–1^ step width. From the ss-DFT simulation (Figure 2-II), the peaks in the calculated spectra can all be assigned to peaks in the experimental spectra (Table S4), indicating the absence of other polymorphic impurities in the DPCC produced by SD.

**Figure 3:**
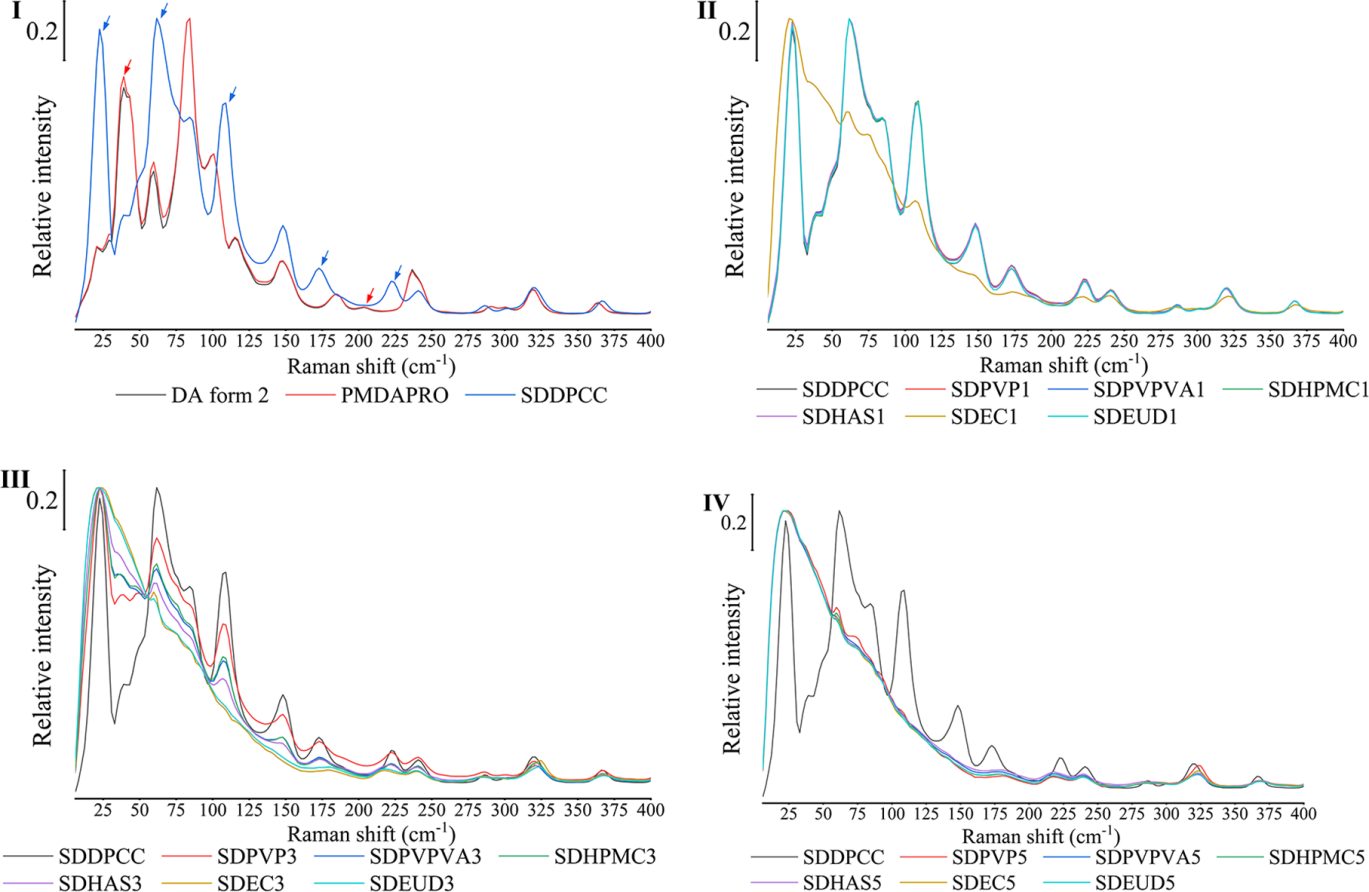
(I) Raman spectra below 400 cm^–1^ collected using the THz-PROBE of DA form II (black spectrum), PMDAPRO (red spectrum), and SDDPCC (blue spectrum). (II) Raman spectra below 400 cm^–1^ collected using the THz-PROBE of SDDPCC (black spectrum), SDPVP1 (red spectrum), SDPVPVA1 (blue spectrum), SDHPMC1 (green spectrum), SDHAS1 (purple spectrum), SDEC1 (brown spectrum), and SDEUD1 (cyan spectrum). (III) Raman spectra below 400 cm^–1^ collected using the THz-PROBE of SDDPCC (black spectrum), SDPVP3 (red spectrum), SDPVPVA3 (blue spectrum), SDHPMC3 (green spectrum), SDHAS3 (purple spectrum), SDEC3 (brown spectrum), and SDEUD3 (cyan spectrum). (IV) Raman spectra below 400 cm^–1^ collected using the THz-PROBE of SDDPCC (black spectrum), SDPVP5 (red spectrum), SDPVPVA5 (blue spectrum), SDHPMC5 (green spectrum), SDHAS5 (purple spectrum), SDEC5 (brown spectrum), and SDEUD5 (cyan spectrum)

In the lower Raman region, the absorption pattern of SDDPCC was clearly different from that of either DA form II or PMDAPRO (Figure 3). DA form II showed distinctive peaks at 39.1 and 203.7 cm^–1^. PROAH and PROHY compared to DA form II and DPCC showed much weaker Raman scatter (Figure S4), so PMDAPRO after normalization showed the same spectra as DA form II (Figure 3). SDDPCC showed characteristic bands at 22.6, 61.7, 109.0, 172.8, and 222.1 cm^–1^. The peaks below 100 cm^–1^ are mostly overlaid between DA form II and SDDPCC, but the difference in the peak intensity is large enough to enable discrimination between API and cocrystal.

The lower Raman region is sensitive to not only polymorphic change but also structural order. Compared to the crystalline DPCC, the ASDs, because of the lack of ordered molecules, present a wide halo peak below 200 cm^–1^ (Figure 3). sCSDs showed characteristically sharp DPCC peaks on the wide halo peak, indicating their semicrystalline solid state. Interestingly, HAS3 and EC1, which were PXRD amorphous (ASDs), also showed small, sharp DPCC characteristic peaks before 100 cm^–1^, indicating that there might be some crystalline cocrystal inside the amorphous matrix.

### 1.7 Solid-State Stability - Cocrystal Dissociation

#### 1.7.1 QPA Model for the Estimation of the Extent of Cocrystal Dissociation

The spectra of different proportions of DPCC in the PMs of DPCC and DA form II are shown in Figure S6. The peak intensity of the distinctive DPCC peak at 22.6 cm^–1^ increased as the proportion of DPCC in the PM increased, while the peak intensity of the DA form II characteristic peak at 39.1 cm^–1^ decreased (Figure S6-I). Moreover, a calibration curve (*R*^2^ > 0.99) (Figure S6-II) was built from the proportion of DPCC in the mix (cocrystal%) and the peak ratio calculated by eq 1. This calibration curve was utilized for a fast estimation of the amount of cocrystal remaining on exposure of cocrystal to an extreme environment (of high RH), and a further discussion regarding the usage of this QPA model is detailed in the Supporting Information.

#### 1.7.2 Cocrystal Dissociation under Accelerated Stability Conditions (40 °C and 95% RH)

The formulations with 1 wt % polymer and the DPCC itself were treated for different periods of time at 40 °C and 95% RH. The analysis by PXRD and cocrystal% estimation using the calibration curve (Figure S6-II) are shown in Figures S8–S14 and 4, respectively. The RMC of each powder after 18 h of exposure is shown in Table S1. The DPCC itself did not show dissociation before 2 h, as there were no Bragg peaks in the PXRD that would indicate the formation of other solid forms. The DPCC exhibited dissociation after 6 h, with 89.2 ± 7.2% cocrystal remaining at 12 h and only 35.7 ± 2.2% cocrystal remaining at 18 h. From the diffractograms at 12 and 18 h, there was an additional characteristic PROHY peak at 8.7° 2θ and a characteristic DA form II peak at 9.3° 2θ (Figure S8). The PROAH might also exist in the dissociated product, but the determination of the PROAH by PXRD was difficult, as the distinctive PROAH peaks at 15.2, 18.1, 18.5, and 19.6° 2θ were overlaid with those of other crystalline phases.

**Figure 4:**
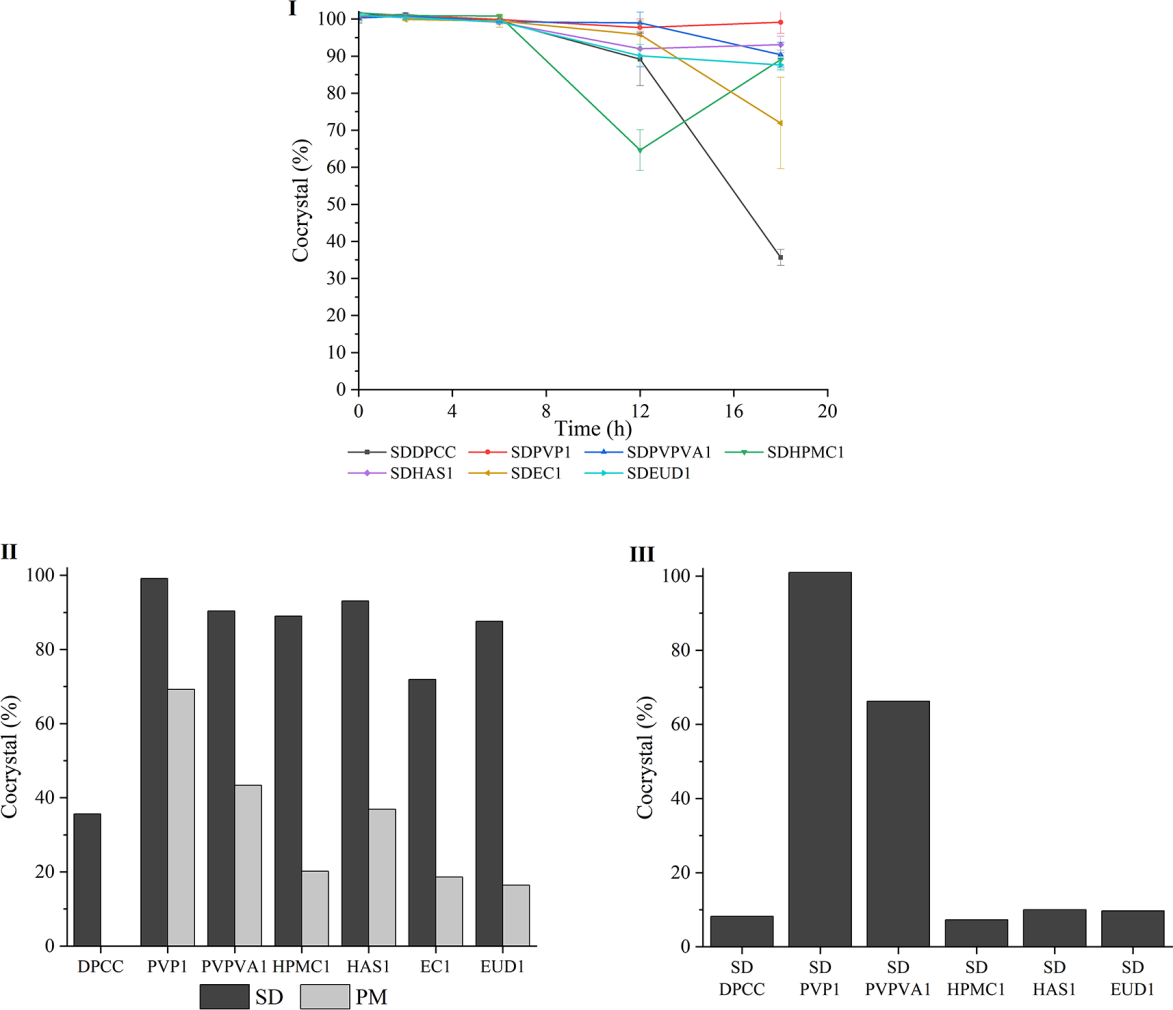
(I) Percentage crystalline DPCC (cocrystal%) of SDDPCC (black profile), SDPVP1 (red profile), SDPVPVA1 (blue profile), SDHPMC1 (green profile), SDHAS1 (purple profile), SDEC1 (brown profile), and SDEUD1 (cyan profile) when stored for 18 h at 40 °C and 95% RH. (II) Percentage crystalline DPCC (cocrystal%) remaining for SDDPCC, SDPVP1, PMPVP1, SDPVPVA1, PMPVPVA1, SDHPMC1, PMHPMC1, SDHAS1, PMHAS1, SDEC1, PMEC1, SDEUD1, and PMEUD1 after being exposed to 40 °C and 95% RH for 18 h. (III) Percentage crystalline DPCC (cocrystal%) remaining for SDDPCC, SDPVP1, SDPVPVA1, SDHPMC1, SDHAS1, and SDEUD1, after exposure to 40 °C and 95% RH for 7 days.

Interestingly, all the CSDs (Table 3) dissociated slower than the crystalline DPCC alone, with SDPVP1 showing the best stability, with 99.1 ± 3.0% cocrystal remaining at 18 h. Dissociation of the cocrystal in all CSDs was seen to begin after 6 h, and SDPVPVA1 (90.4 ± 3.3%), SDHPMC1 (89.0 ± 2.6%), SDHAS1 (93.1 ± 2.2%), and SDEUD1 (87.6 ± 1.5%) still had around 90% cocrystal remaining at 18 h, of which the proportion of cocrystal in SDHPMC1 declined dramatically to 64.6 ± 5.5% at 12 h but recovered back to 89.0 ± 2.6% at 18 h. This “rebound” effect may be because of a complicated equilibrium between the polymer, each crystalline phase, the relative humidity, the water absorbed by solids (Table S1), and the DA and PRO dissolved in the sorbed water.^8,11,60−63^ SDEC1, the only ASD involved in the stability study, recrystallized fully (99.90 ± 0.39%) to cocrystal at 2 h and suddenly started to dissociate after 12 h. There was only 71.92 ± 12.33% cocrystal remaining at 18 h, indicating SDEC1 also has a better stability than SDDPCC (35.7 ± 2.2% cocrystal remained at 18 h).

The stability performance at 18 h of the PMs with the same composition as that of CSDs was also analyzed (Figures 4-II, S15). 1 wt % of PVP (69.2% cocrystal remaining) and PVPVA (43.4% cocrystal remaining) blended with DPCC provided much stronger protection against cocrystal dissociation compared to the DPCC alone (35.7% cocrystal remaining). 1 wt % of HAS (36.9% cocrystal remaining) blended with cocrystal seemed to have no effect on the dissociation of cocrystal, but 1 wt % of HPMC (20.2% cocrystal remaining), EC (18.6% cocrystal remaining), and EUD (16.4% cocrystal remaining) appeared to accelerate the dissociation. Hence, PVP and PVPVA are the most suitable excipients for protection of DPCC dissociation in a high-humidity environment. Moreover, all CSDs demonstrated enhanced stability compared to PMs, even for those spray-dried solid dispersions that contained polymers that could accelerate the dissociation of the cocrystal when physically mixed with it. Thus, all CSDs were further analyzed over a 7 day stability study (Figures 4-III, S16) under the same environmental conditions. Surprisingly, SDPVP1 and SDPVPVA1 could still provide protection to cocrystal against high humidity for 7 days, and there was no dissociation of the cocrystal in SDPVP1 and only partial dissociation in SDPVPVA1. For other CSDs and the DPCC itself, only around 8% cocrystal was left after 7 days. Preparing the cocrystals by co-spray-drying the API and coformer with polymers is clearly advantageous to the physical stability of the cocrystals.

### 1.8 Monitoring Cocrystal Stability When Exposed to Liquid Medium

From the solid-state stability study at 95% RH, SDPVP1 and SDPVPVA1 maintained a higher cocrystal% (undissociated) compared to other dispersions and SDDPCC. As for the next step, it was decided to investigate the stability of DPCC in SDPVP1 and SDPVPVA1 dispersions upon exposure to dissolution medium. The advantage of a higher dissolution rate should be retained for a longer period of time if the cocrystal can maintain its undissociated form for a longer period of time.

#### 1.8.1 Selecting Appropriate Raman Region

**Figure 5:**
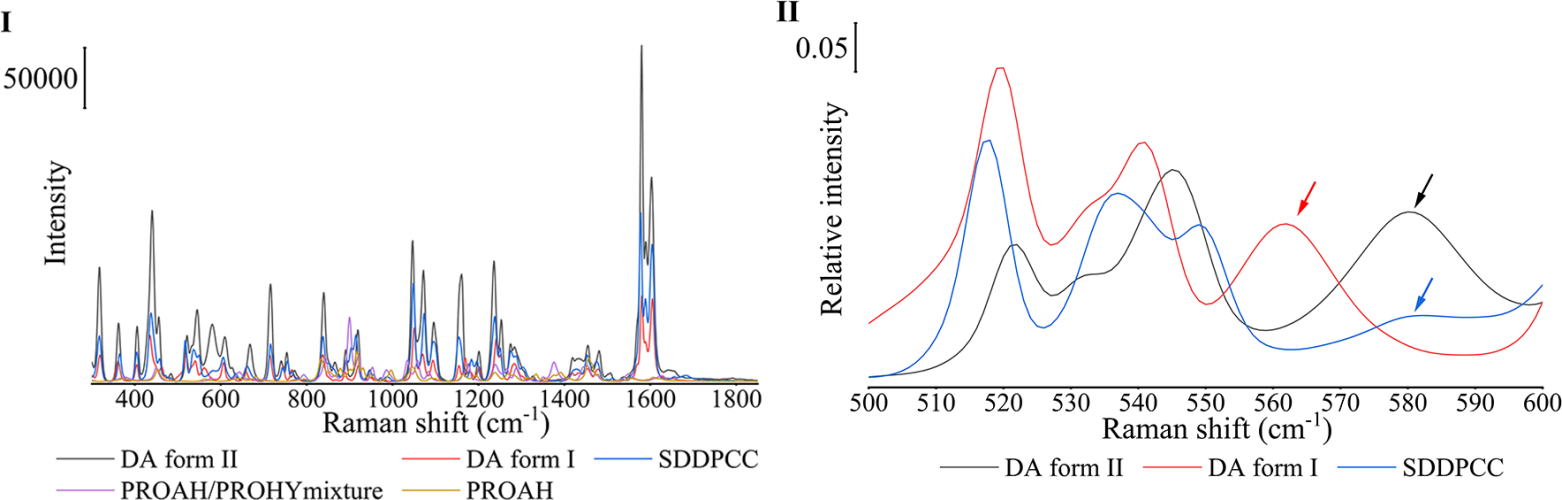
(I) Raman spectra collected by the PhAT probe of DA form II (black spectrum), DA form I (red spectrum), SDDPCC (blue spectrum), PROAH/PROHY mixture (purple spectrum), and PROAH (brown spectrum). (II) Raman spectra (normalized based on the peak intensity at 1578 cm^–1^) were collected by the PhAT probe of DA form II (black spectrum), DA form I (red spectrum), and SDDPCC (blue spectrum).

The PhAT probe was used to collect spectra every 30 s, while DPCC was covered by liquid medium to examine the stability of SDDPCC, SDPVP1, and SDPVPVA1, as it could generate high-signal data in a short period. The spectra of all related crystalline phases that DPCC might be able to dissociate to are shown in Figure 5-I. After the normalization of the spectra of DA form I, DA form II, and DPCC to the peak intensity at 1578 cm^–1^, a clear spectra difference between DPCC and DA polymorphs can be observed between 500 and 600 cm^–1^ (Figure 5-II). DA form I and DA form II showed a medium peak intensity at 560 and 580 cm^–1^ (as shown by the red and black arrows in Figure 5-II) respectively, but DPCC presented a much weaker Raman signal at these Raman shifts (as shown by a blue arrow in Figure 5-II). Thus, during this stability study, the peak relative intensity at 560 and 580 cm^–1^ to the peak intensity at 1578 cm^–1^ was monitored to track the possible dissociation pathway to the DA polymorphs. PROAH and the PROAH/PROHY mixture revealed a weak Raman scatter in the Raman regions between 550 and 600 cm^–1^. Furthermore, most PRO molecules dissociated from cocrystal were immediately dissolved in the liquid medium (as observed in Raman spectra, data not shown, and QPA of powder by PXRD), as PRO is highly soluble in both pH 6.8 phosphate buffer and water.^32,64^ Thus, the PROAH and PROAH/PROHY mixture should not significantly affect monitoring of the dissociation process.

#### 1.8.2 Monitoring Cocrystal Stability When Exposed to pH 6.8 Phosphate Buffer

**Figure 6:**
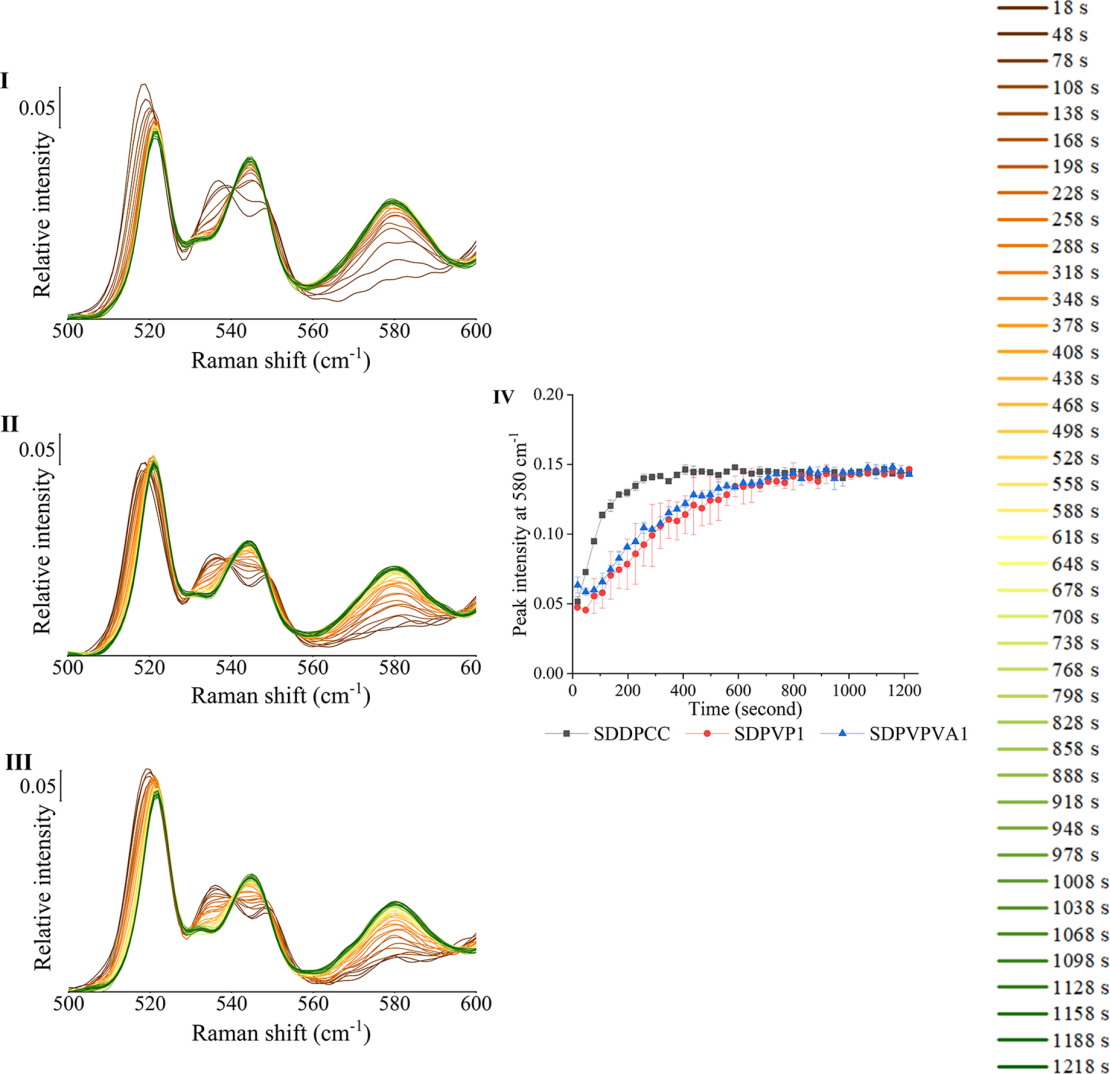
Raman spectra of SDDPCC (I), SDPVP1 (II), and SDPVPVA1 (III) exposed to pH 6.8 phosphate buffer. The time points at which the spectra were collected are shown on the right of the Figure. (IV) Raman peak relative intensity at 580 cm^–1^ of SDDPCC (black profile), SDPVP1 (red profile), and SDPVPVA1 (blue profile) exposed to pH 6.8 phosphate buffer.

SDDPCC, SDPVP1, and SDPVPVA1 were exposed to pH 6.8 phosphate buffer for 20 min, and the spectra were collected. The change in the relative peak intensity at 580 cm^–1^ with time is shown in Figure 6. The QPA of the solid (by PXRD Rietveld refinement) after 20 min of exposure to pH 6.8 phosphate buffer is shown in Table 4. There was no cocrystal remaining (Table 4) in SDDPCC, SDPVP1, or SDPVPVA1 after 20 min of exposure to pH 6.8 phosphate buffer, suggesting that the cocrystal completely dissociated in each solid. Also, the proportion of DA form I and DA form II remaining in each solid after 20 min of exposure did not differ significantly. Despite the fact that all solids completely dissociated to an identical amount of DA form I and DA form II within 20 min, the time each solid took to achieve complete dissociation varied. The spectrum of each solid showed that after 20 min of exposure, the peak intensity at 580 cm^–1^ dramatically increased, and there is no peak at 560 cm^–1^, indicating that cocrystal primarily dissociated to DA form II, consistent with the QPA estimation (Table 4). Nonetheless, the spectrum of SDDPCC was only changed slightly after 300 s, and the peak intensity at 580 cm^–1^ increased in the first 400 s and then leveled out. However, for both SDPVP1 and SDPVPVA1, it took 800 s until the spectra changed, and the peak intensity at 580 cm^–1^ for both dispersions did not reach the same level as the 400 s exposure of SDDPCC until 800 s (Figure 6). Hence, although all solids were completely dissociated in the 20 min study, it took longer for SDPVP1 and SDPVPVA1 to reach complete dissociation than SDDPCC, indicating that the cocrystal dissociates more slowly when incorporated in a CSD when exposed to pH 6.8 phosphate buffer.

**Table 4:** QPA Estimation of SDDPCC, SDPVP1, and SDPVPVA1 after 20 min of Exposure of pH 6.8 Phosphate Buffer

Solid form/dispersions	QPA by Rietveld refinement	Rwp	GOF
SDDPCC	9.0% DA form I + 91.0% DA form II	11.69	2.09
SDPVP1	5.1% DA form I + 94.5% DA form II	11.18	2.22
SDPVPVA1	4.2% DA form I + 95.8% DA form II	10.18	2.16

#### 1.8.3 Monitoring Cocrystal Stability When Exposed to Water

**Figure 7:**
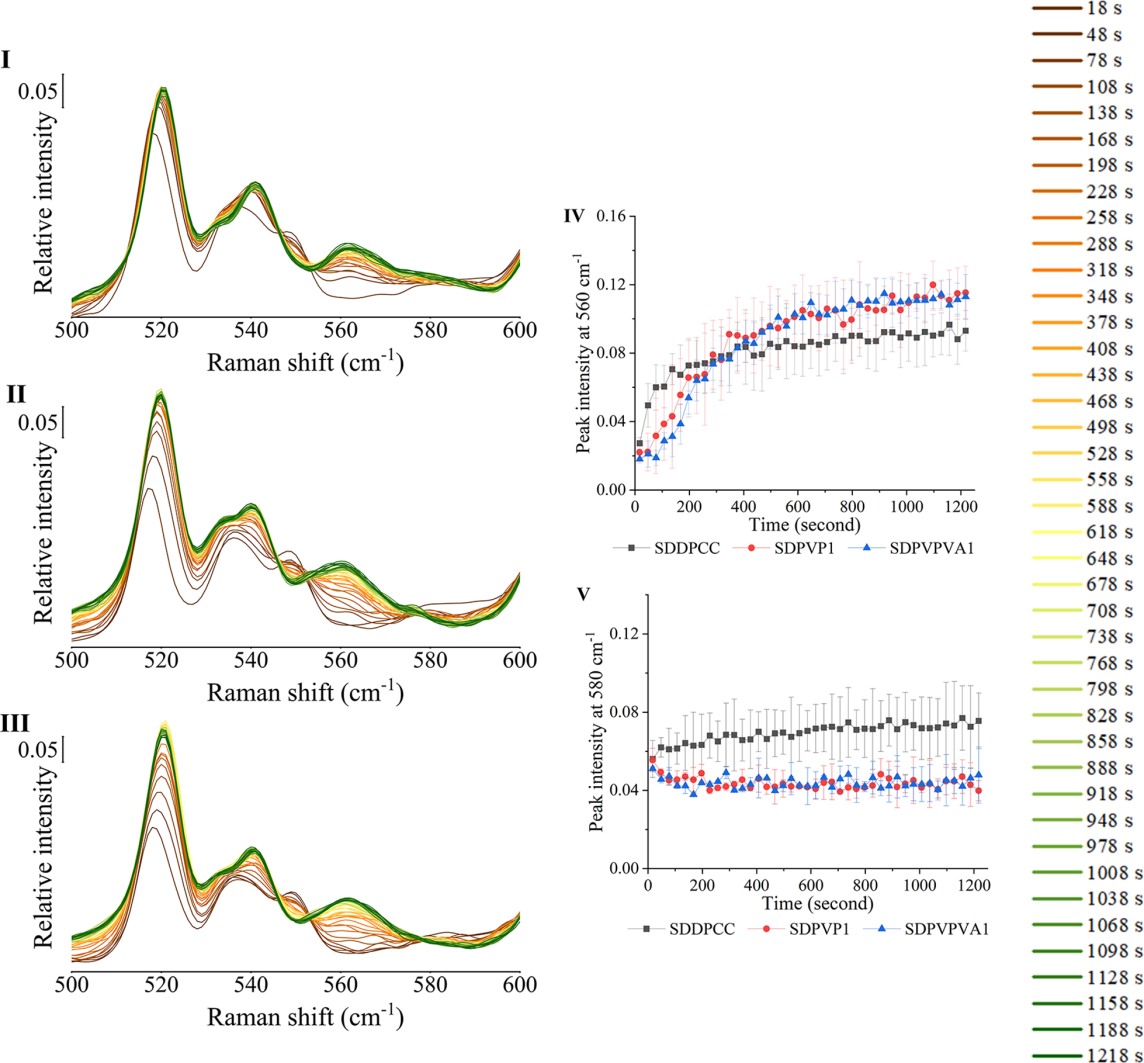
Raman spectra of SDDPCC (I), SDPVP1 (II), and SDPVPVA1 (III) exposed to water. The time points at which the spectra were collected are shown in the right of the Figure. (IV) Raman peak relative intensity at 560 cm^–1^ of SDDPCC (black profile), SDPVP1 (red profile), and SDPVPVA1 (blue profile) exposed to water. (V) Raman peak relative intensity at 580 cm^–1^ of SDDPCC (black profile), SDPVP1 (red profile), and SDPVPVA1 (blue profile) exposed to water.

SDDPCC, SDPVP1, and SDPVPVA1 were also exposed to water for 20 min, and the spectra were collected. The change in relative peak intensities at 560 and 580 cm^–1^ with time is shown in Figure 7. The QPA of the solid after 20 min of exposure to water is shown in Table 5. QPA estimation by PXRD (Table 5) indicated that the cocrystal remaining in the three samples after 20 min of exposure to water was similar, at around 13%, which is different from pH 6.8 phosphate buffer, which resulted in complete dissociation of the cocrystal in the three samples by 20 min. Moreover, unlike the situation with phosphate buffer, where the three samples dissociated mainly to DA form II, the three samples dissociated to both DA form I and DA form II when exposed to water. Additionally, there was more DA form I and less DA form II remaining in SDPVP1 and SDPVPVA1 compared to SDDPCC. Since all three solids dissociated in water to both DA polymorphs, in different proportions, it is difficult to compare the dissociation rate directly from the peak intensity of DA polymorph characteristic peaks at 560 and 580 cm^–1^. However, from QPA estimation by PXRD, although the constituent fraction of the remaining solid at the end time point was complex in the three samples, all samples contained the same cocrystal content, at around 13%. Therefore, as the cocrystal dissociates, the closer the spectrum is to the spectrum of the end time point, which is indicative of 13% cocrystal, the lower the cocrystal content. For SDDPCC, its spectrum dramatically changed from the beginning to 50 s, and the spectrum at 50 s was already similar to its spectrum at the end time point, indicating most SDDPCC was completely dissociated in the 50 s. Compared to SDPVP1 and SDPVPVA1, the trend of cocrystal spectrum converting to the spectrum at the end time point was clearly slower than for SDDPCC, where both SDPVP1 and SDPVPVA1 took around 200 s to have a spectrum which was close to the spectrum at the end time point.

**Table 5:** QPA Estimation of SDDPCC, SDPVP1, and SDPVPVA1 after 20 min of Exposure of Water

Solid form/dispersions	QPA by Rietveld refinement	Rwp	GOF
SDDPCC	33.7% DA form I + 21.1% DA form II + 12.6% DPCC + 32.6% PROHY	10.95	2.84
SDPVP1	65.9% DA form I + 7.6% DA form II + 13.3% DPCC + 13.2% PROHY	11.35	1.87
SDPVPVA1	62.6% DA form I + 14.9% DA form II + 12.8% DPCC + 9.7% PROHY	12.54	2.47

**Figure 8:**
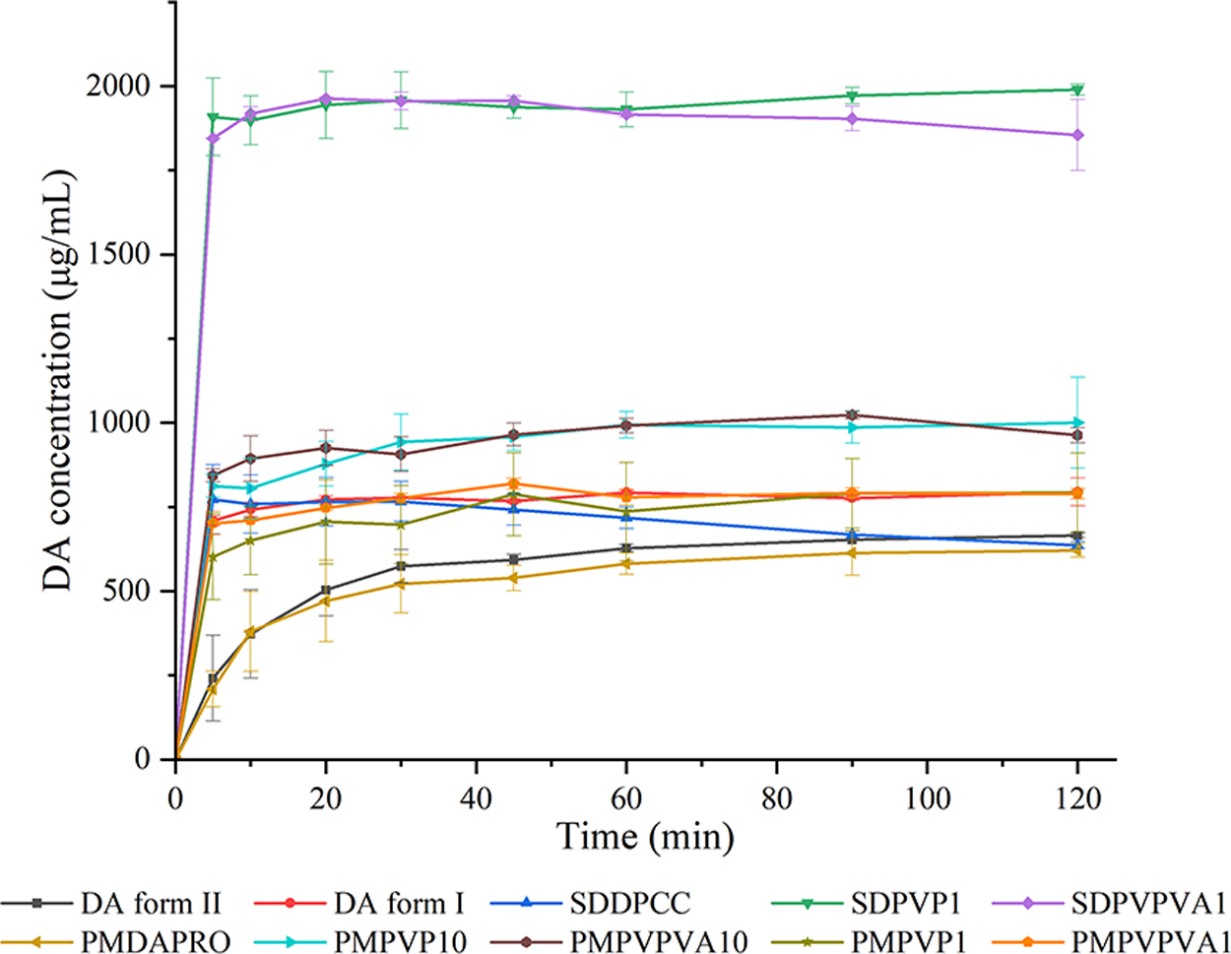
Dynamic solubility in pH 6.8 phosphate buffer of DA form II (black profile), DA form I (red profile), SDDPCC (blue profile), SDPVP1 (green profile), SDPVPVA1 (purple profile), PMDAPRO (light brown profile), PMPVP10 (cyan profile), PMPVPVA10 (dark brown profile), PMPVP1 (army green profile), PMPVPVA1 (orange profile).

In summary, although the cocrystal% remaining after 20 min exposure to water is similar in all three samples, the dissociation rate of SDPVP1 and SDPVPVA1 was clearly slower than SDDPCC at the beginning. Furthermore, it seems that once the cocrystal content was close to 13%, the dissociation suddenly became much slower in water. This is probably because there is an equilibrium between the undissolved and dissolved components, which hinders the further dissociation of the remaining cocrystal content. The reasons why the cocrystal stability is different in different media are probably due to the differences in medium composition, cocrystal solubility (Figures 8 and S26), and pH of the medium after dissolution (Table S7), resulting in different complex equilibriums between the solid components, dissolved API and coformer, and the ions in the medium.

### 1.9 Dynamic Solubility Study

The solubility of DA form II and DA form I in phosphate buffer was 665.61 ± 6.51 and 794.62 ± 41.14 μg/mL, respectively, at 2 h (Figure 8). The PMDAPRO demonstrated a similar dynamic solubility to DA form II and reached a solubility of 621.6 ± 20.30 μg/mL at 2 h. There is no statistical difference (*p* > 0.05) in the solubility after 2 h between DA form II and PMDAPRO, suggesting that the PRO does not influence the solubility of DA form II. SDDPCC reached its highest DA concentration of 772.53 ± 103.21 μg/mL at 5 min and decreased slightly thereafter to 636.79 ± 10.10 μg/mL at 2 h, which was close to the solubility value of DA form II. The solubility profile indicates that DA reached supersaturation at 5 min but was unable to maintain a supersaturation state, resulting in the precipitation of DA form II (Table 6) and consequently a decrease in DA concentration in solution. Interestingly, SDPVP1 and SDPVPVA1 retained a solubility of DA of around 1900 μg/mL over 2 h, which was roughly 3-fold that of SDDPCC or DA form II. The remaining solid contained not only DA form II but also a small amount of DA form I (around 7.6%) (Table 6).

**Table 6:** QPA Estimation after 2 h of Dynamic Solubility Study in pH 6.8 Phosphate Buffer^a^

Solid form/dispersions	QPA by Rietveld refinement	Rwp	GOF
DA form II	100% DA form II*	N/A*	N/A*
DA form I	95.8% DA form I + 4.2% DA form II	16.11	4.18
SDDPCC	100% DA form II*	N/A*	N/A*
PMDAPRO	100% DA form II*	N/A*	N/A*
SDPVP1	7.8% DA form I + 92.2% DA form II	11.81	4.84
SDPVPVA1	7.5% DA form I + 92.5% DA form II	14.34	4.65
PMPVP1	4.3% DA form I + 95.7% DA form II	15.60	4.38
PMPVPVA1	4.5% DA form I + 95.5% DA form II	12.28	2.10
PMPVP10	8.4% DA form I + 91.6% DA form II	13.39	2.80
PMPVPVA10	7.1% DA form I + 92.9% DA form II	11.89	2.15

a * indicates diffractograms that only show one phase, so Rietveld refinement was not necessary. N/A: not applicable.

In order to understand the mechanism of enhancement of the solubility by the solid dispersions, PMs between SDDPCC and different amounts of polymer were prepared (PMPVP1, PMPVPVA1, PMPVP10, and PMPVPVA10). Although there was no statistical difference (*p* > 0.05) in the DA solubility between PMs that contained 1 wt % of polymer (PMPVP1 and PMPVPVA1) and SDDPCC at 2 h, the precipitation phenomenon of these PMs was not as obvious as for the SDDPCC, where the PMs always maintained the concentration of DA above 700 μg/mL after 10 min. Nevertheless, for the PMs that contained 10 wt % of polymer (PMPVP10 and PMPVPVA10), a higher DA solubility was attained after 2 h than for SDDPCC, with the PM containing PVP attaining the highest concentration, of 1001.00 ± 135.09 μg/mL, at 2 h, and the PM with PVPVA attaining the highest concentration, of 1023.33 ± 11.04 μg/mL, at 1.5 h. There was also no obvious precipitation observed for both PMs with the higher concentration of polymer. Thus, the introduction of a small amount of PVP or PVPVA could prevent the crystallization of API from pH 6.8 phosphate buffer when the concentration of DA exceeded its saturation. Polymers maintaining a supersaturation state of API dissolved from cocrystal were also reported by Alhalaweh et al.^13^

Since PVP and PVPVA did not present an obvious solubilization effect, the reason that the two CSDs containing 1 wt % polymer reached extraordinarily high solubility might because the two dispersions can maintain the undissociated cocrystal solid state for much longer than DPCC on its own in pH 6.8 phosphate buffer (Figure 6), which is beneficial to the solubilization effect by SDDPCC, leading to more DA in the dissolved state. Furthermore, the dissolved polymer from the dispersions also assisted in preventing precipitation from the supersaturation state, so even though the two dispersions resulted in the saturation level being exceeded by around 3-fold, the dissolved DA was not precipitated. Additionally, SDPVP1 and SDPVPVA1 also attained a higher solubility than SDDPCC and DA polymorphs in water (Figure S26, Table S6), probably also because an undissociated cocrystal solid could be maintained in the medium for longer, as was observed in pH 6.8 phosphate buffer.

### 1.10 Mechanism of Cocrystal Physical Stability Enhancement

As discussed in previous sections, the physical stability of SDPVP1 and SDPVPVA1 is superior to that of SDDPCC. The superior physical stability of the solid dispersions retards dissociation of the cocrystal when solids are exposed to liquid, which conveys a positive impact on solubility enhancement. In light of this, it is important to determine the mechanism by which the polymers can physically protect the cocrystal (from dissociation), which may offer advantages for the formulation and development of cocrystals.

#### 1.10.1 Computational Prediction

**Figure 9:**
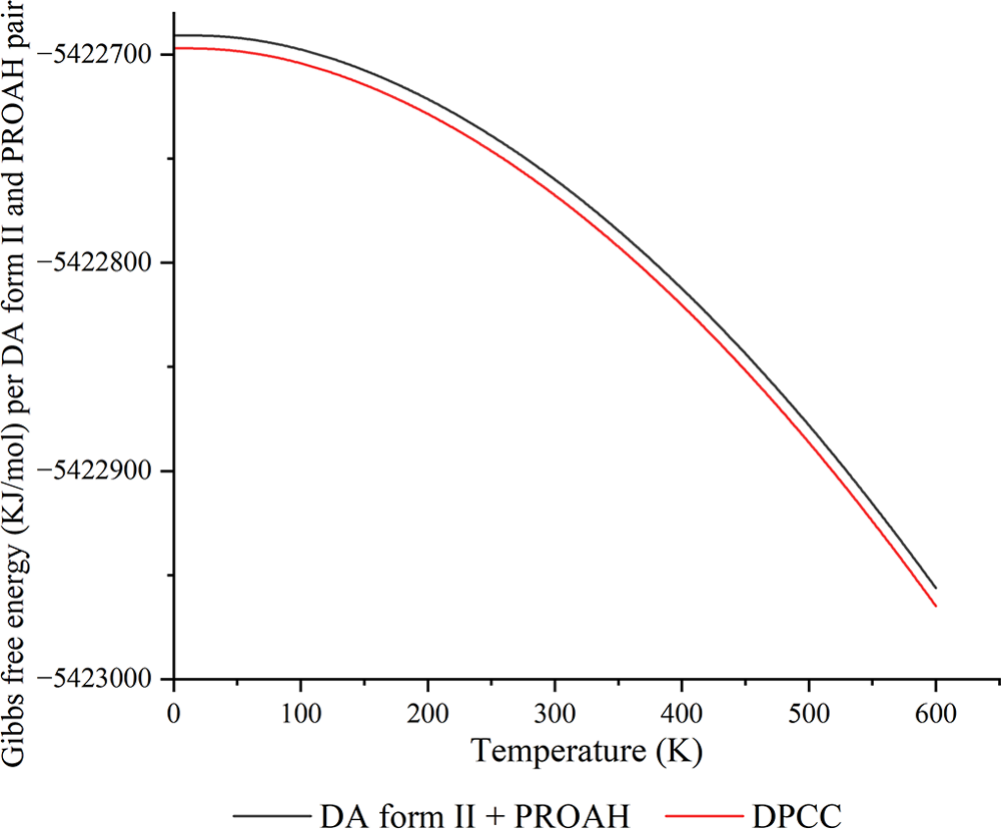
Gibbs free energies (kJ/mol) of DPCC and the sum of pure component crystals.

The relatively narrow peak widths in the THz-Raman spectra at room temperature and the lack of significant peak shifting with sample cooling (Figure 2) suggest that the large-amplitude lattice vibrations of the cocrystal exist in a near-harmonic potential energy surface. This is confirmed by the excellent reproduction of the observed THz-Raman spectrum by THz-BENCH (Figure 2) by ss-DFT harmonic normal-mode analysis. A harmonic potential governing the intermolecular interactions within the cocrystal suggests that the solid is energetically stable, as strongly anharmonic vibrations tend to be related with broad and shallow potential energy surfaces. As a test of this concept, the calculated Gibbs free energies of pure crystals of DA form II and PROAH have been compared to the Gibbs free energy of DPCC. It was found that the cocrystal is thermodynamically preferred across all temperatures considered, with Δ*G* = −7.5 kJ/mol per diclofenac–proline pair in the cocrystal at 295 K (Figure 9). The energetic advantage of cocrystal formation as compared to the pure components indicates that the cocrystal should exhibit long-term stability and that its dissociation under laboratory conditions may be driven by outside factors, such as water-mediated dissociation.

#### 1.10.2 Gelling *In Situ* of Polymers Protecting Cocrystal Physically

SDDPCC is physically unstable at 40 °C/95% RH, and there is a large amount of water sorption (RMC = 12.20 ± 0.88%) after 18 h exposure (Table S1). As a result, the driving force for the dissociation of DPCC is water-mediated dissociation. As previously reported, moisture sorption by cocrystals enhances the mobility of the surface molecules and facilitates the dissociation transformation or results in hydrate phase formation of API or coformer, enabling dissociation.^11,65,66^

From Figure 4-I, all CSDs afforded a short-term protection to the cocrystal from dissociation and showed much better stability than the PMs with same proportions of polymer and cocrystal as CSDs, indicating that CSDs prepared by spray drying a homogeneous API–coformer–polymer ethanolic solution might provide mixing at a molecular level between cocrystal molecules and polymer chains, so that polymer chains are molecularly dispersed in the cocrystal molecules’ matrix and more effectively slow down the cocrystal dissociation compared to PMs. Specific intermolecular interactions, such as hydrogen bonding, between cocrystal and polymer might also be a factor contributing to the stabilizing effect provided by CSDs; however, FTIR (Figures S17, S18) and Raman analysis (Figures S2-II, 3-II) did not reveal any evidence of peak shifts that would indicate such interactions between cocrystal and polymer. Additionally, SEM indicates that SDDPCC, SDPVP1, and SDPVPVA1 all crystallized in a monoclinic-shaped powder form (Figures S22–S24). No significant morphology differences were seen between the three samples, and there is no statistically significant difference (*p* > 0.05) in the d10, d50, and d90 parameters between the three samples (Figures S19–21, Table S5). Therefore, it appears that differences in dynamic solubility and solution-mediated phase transformation of the cocrystals between DPCC and the two CSDs are not affected by morphology or particle size.

From a molecular-level perspective, absorption of water by powder should occur initially at the surface of the powder. The water molecules could then penetrate through the layer of powder and affect molecules in the interior.

From previous reports, polymers could slow cocrystal dissociation in two ways. On one hand, polymer could absorb water and reduce the bulk moisture content to which the cocrystal is exposed, slowing down water-mediated dissociation.^61^ On the other hand, polymers could undergo self-gelation after water sorption, which could provide a physical barrier for diffusion of water to the cocrystal, providing protection against high humidity.^61,67^

However, in our study, the weight ratio between polymer and cocrystal molecules is 1:100 w/w instead of 1:1 w/w, as was the case in the study of Suzuki et al.^61^ or 1:20 w/w, as in the study of Ross et al.^67^ Therefore, the amount of polymer used in our CSDs should not sequester enough water to prevent the cocrystal from dissociation, and the “sequester effect” most likely does not dominate the protecting process.

We hypothesize that since spray drying can provide molecular-level mixing between cocrystal molecules and polymer chains, the polymer chains dispersed close to the surface or on the surface of CSD particles could swell and undergo gelling *in situ*, providing a physical barrier to moisture diffusing as far as the cocrystal molecules to the interior of particles, further from the surface. An *in situ* gelling process was previously observed by Ross et al. for dispersions of indomethacin–saccharin cocrystals in HPMC and Neusilin and was determined to be the main reason for the superior physical stability of these systems in terms of preventing cocrystal dissociation.^67^ Even though the cocrystal molecules on the surface might dissociate, the physical barrier provided by the polymer will slow water penetration to prevent bulk cocrystal dissociation. Furthermore, the water that penetrates the first barrier of the polymer gel will cause other polymer chains close to the first barrier to undergo *in situ* swelling and gelling and become a further barrier to moisture ingress.

In our study, SDPVP1 and SDPVPVA1 provided the best and second-best protection to cocrystal dissociation. Both PVP and PVPVA are also the most hygroscopic polymers used in our study (Table S2). Compared to other polymers, PVP and PVPVA powders underwent an obvious, visually observed, *in situ* gelling on exposure of the polymer powder to 95% RH at 40 °C (Figure S25). All PVP/PVPVA powders turned into viscous gels, which could be observed visually (Figure S25) and which, we hypothesize, could serve as a superior physical barrier against high humidity in a mixed system with cocrystals, compared to other polymers. The superior barriers of PVP and PVPVA gel provided protection to DPCC by intimate mixing of PVP/PVPVA in the SDDPCC system, leading to a low moisture content of SDPVP1 and SDPVPVA1 (Table S1) after 18 h of exposure to 95% RH (Figure 4-II).

In the polymer hygroscopicity study, HPMC, HAS, EC, and EUD were less hygroscopic than PVP and PVPVA (Table S2) and visually remained as powders (rather than gels) when exposed to 95% RH (Figure S25). The RMCs for the CSDs containing HPMC, HAS, EC, and EUD after 18 h at 95% RH ranged from 3.99 to 10.28%, which are lower than SDDPCC (12.20% cocrystal remained) while also having a higher cocrystal content (87–93% cocrystal remaining) than SDDPCC (35.7% cocrystal remaining). These observations indicate that the polymer chains without/with nonobvious swelling or gelation could also act as a barrier to slow down water penetration to the bulk powder. However, the barriers provided are not as strong as the gels of PVP and PVPVA, so their solid dispersions cannot provide long-term stability.

Cocrystal dissociation in a high-humid environment or in dissolution medium is a complicated process, which involves the transformation between solid cocrystal/API/coformer and liquid API/coformer and, potentially, between different solid-state forms. Furthermore, the amount of water sorption, solubilities of the cocrystal/API/coformer, particle size of the powder, and the dissolution rate of the cocrystal/API/coformer in the absorbed water or dissolution medium can also affect the cocrystal dissociation process. With different excipients involved, the dissociation process will be even more complex.

Given the low percentage of polymer present in the CSDs, our hypothesis that the polymer gelling effect provides a barrier that protects the cocrystal from dissociation is more credible if the polymer is distributed on the surface of particles, providing a protective barrier to the cocrystal located in the interior. Further work (e.g., using ICP-MS) is required to try to elucidate the polymer–cocrystal distributions at the particulate level, with a view to better understanding the polymer protection from the dissociation effect.

## Conclusions

The contributions from the present study provide a greater understanding of the preparation of CSDs (comprised of cocrystal and polymer) and the physical stability enhancement of cocrystals in terms of protection from dissociation provided by CSDs.

Spray drying has been demonstrated to be a viable technique for the one-step *in situ* manufacture of CSDs composed of cocrystal and polymer, with the type and loading of the polymer impacting the physicochemical properties of the solid dispersions generated.

CSDs generated *in situ* with even 1 wt % polymer (as a percentage of cocrystal) have the potential to enhance physical stability and improve the solubility and dissolution characteristics of the API. CSDs with PVP and PVPVA provided a longer-term physical stability to the cocrystal compared to other CSDs and the cocrystal on its own.

The mechanism by which CSDs protect the cocrystal from dissociation may be related to the polymer and cocrystal in the CSD achieving molecular-level mixing, whereby the polymer chains after gelling *in situ* slow down water penetrating to the bulk cocrystal molecules, which prevents the bulk cocrystal molecules from interacting with water, thereby slowing down the water-mediated dissociation.

## Supporting Information Available

Detailed information on the asymmetric unit of the diclofenac acid–l-proline cocrystal, residual moisture/solvent content of the samples before/after stability study, Raman analysis of each sample by means of the PhAT probe, low-frequency Raman spectra of the cocrystal, API, coformer, and polymers utilized in this study, QPA model for estimation of cocrystal content, PXRD diffractograms of each sample from the stability study, FTIR spectra of each crystalline solid dispersion, particle size analysis data, SEMs, imaging of polymer after the stability study, results of the dynamic solubility study in water, pH measurements after the dynamic solubility study (PDF)
