# Supplementary material for: In Situ Cocrystallization via Spray Drying with Polymer as a Strategy to Prevent Cocrystal Dissociation
Source: Mol Pharm. 2023 Aug 18;20(9):4770–85. doi: 10.1021/acs.molpharmaceut.3c00564 (PMC10481393; doi:10.1021/acs.molpharmaceut.3c00564)
Supplement: Supplementary file 1 — mp3c00564_si_001.pdf [file mp3c00564_si_001.pdf]

Supporting information

In-situ co-crystallization via spray-drying with polymer as a strategy to prevent cocrystal dissociation

Detailed information on the asymmetric unit of the diclofenac acid-L-proline cocrystal, residual moisture/solvent content of the samples before/after stability study, Raman analysis of each sample by means of the PhAT probe, low frequency Raman spectra of the cocrystal, API, coformer, and polymers utilized in this study, QPA model for estimation of cocrystal content, PXRD diffractograms of each sample from the stability study, FTIR spectra of each crystalline solid dispersion, particle size analysis data, SEMs, imaging of polymer after the stability study, results of the dynamic solubility study in water, pH measurements after the dynamic solubility study.

ShiZhe Shao[a, b], Michael W. Stocker [a, b, e], Salvatore Zarrella[c], Timothy M. Korter[c], Abhishek Singh[d], Anne Marie Healy[a, b] *

[a] School of Pharmacy and Pharmaceutical Sciences, Trinity College Dublin, Dublin, D02 PN40, Ireland
[b] SSPC, The SFI Research Centre for Pharmaceuticals
[c] Department of Chemistry, Syracuse University, 1-014 Center for Science and Technology, Syracuse, New York, 13244, United States
[d] Janssen Pharmaceutica NV, Beerse, 2340, Belgium
[e] School of Chemical and Bioprocess Engineering, University College Dublin, Dublin,, D04 V1W8, Ireland

* Corresponding Author
Tel: +353-1-8961444
Email: healyam@tcd.ie

Table of Contents

1.1 ASYMMETRIC UNIT OF DPCC 3

1.2 TGA ANALYSIS 3

1.3 RAMAN ANALYSIS IN FINGERPRINT REGION 4

1.4 RAMAN ANALYSIS IN LOW-FREQUENCY REGION 6

1.5 QPA MODEL IN LOW-FREQUENCY RAMAN REGION 8

1.6 PXRD ANALYSIS 11

1.7 FTIR ANALYSIS 16

1.8 PARTICLE SIZE ANALYSIS 17

1.9 SEM 19

1.10 IMAGING OF POWDER IN BULK 21

1.11 DYNAMIC SOLUBILITY IN WATER 22

1.12 PH MEASUREMENT 23

1.13 REFERENCES 23

1.1 Asymmetric unit of DPCC

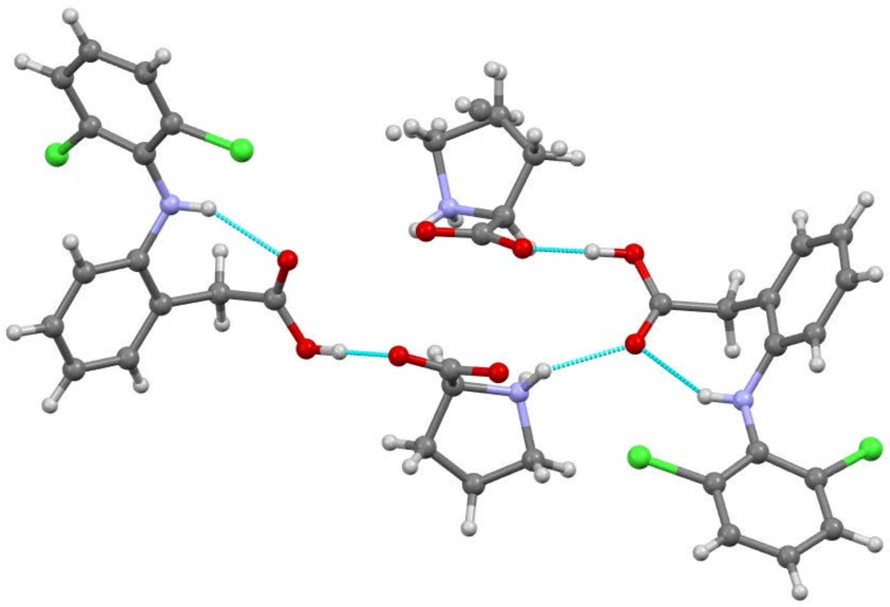

Figure S1. Asymmetric unit of DPCC (CCDC Refcode: RETNEM01)

1.2 TGA analysis

Table S1. RSC of SDDPCC and solid dispersions, and RMC of the solids after 18 h at 40 °C and 95% RH, determined by TGA. N/A: not applicable.

Solid	***RSC (%)***	***RMC (%) at 18 h of 40 °C and 95% RH***	***RMC (%) at 7 days of 40 °C and 95% RH***
SDDPCC	0.12 ± 0.07%	12.20 ± 0.88%	30.31 ± 3.14%
SDPVP1	0.40 ± 0.02%	0.47 ± 0.12%	1.36 ± 0.19%
SDPVPVA1	0.18 ± 0.05%	0.29 ± 0.01%	28.06 ± 0.29%
SDHPMC1	0.36 ± 0.30%	9.44 ± 1.41%	28.72 ± 2.03%
SDHAS1	0.15 ± 0.05%	7.76 ± 0.46%	31.08 ± 0.74%
SDEC1	0.85 ± 0.02%	10.28 ± 1.30%	N/A
SDEUD1	0.32 ± 0.01%	3.99 ± 0.70%	28.41 ± 1.81%

Table S2. Polymer hygroscopicity study. RMC was determined by TGA. N/A: not applicable.

Polymer	*RMC (%) at 18 h of 40 °C and 95% RH*	*RMC (%) at 2 h of 40 °C and 95% RH*
PVP	N/A, self-gelation	17.80 ± 1.12%
PVPVA	N/A, self-gelation	10.68 ± 0.30%
HPMC	9.81 ± 0.29%	4.94 ± 0.32%
HAS	4.61 ± 0.64%	2.83 ± 0.04%
EC	2.43 ± 0.12%	1.08 ± 0.06%
EUD	12.30 ± 0.41%	7.43 ± 0.04%

1.3 Raman analysis in fingerprint region

The Raman spectra of DA form II, PROAH, PMDAPRO, SDDPCC, and the spray dried solid dispersions are displayed in Figure S2. Because the PhAT Raman is a fast and non-destructive technique that can produce a high signal-to-noise ratio in the fingerprint region, it was initially chosen to investigate whether the fingerprint region can be used to distinguish between DPCC and the equivalent physical mix, i.e. PMDAPRO. The Raman spectra of PMDAPRO did not present a very clear difference from the DPCC spectra, but some minor differences between PMDAPRO and DPCC could still be seen (Table S3). The difference in peak positions between PMDAPRO and DPCC was very small, and almost all peaks in the two spectra overlayed, making the Raman fingerprint region more useful for simple qualitative purposes and less suited to conducting quantitative analysis between DA form II and DPCC.

All CSDs presented the same Raman spectra as SDDPCC (Figure S2). Moreover, all ASDs presented similar spectra, and all sCSDs showed an intermediate spectrum between CSDs and ASDs. The ASDs’ spectra were a ‘smooth version’ of the CSDs/PMDAPRO spectra, especially in the Raman region from 732 to 804 cm^{-1}, from 875 to 967 cm^{-1}, and from 1260 to 1332 cm^{-1}. ASDs presented characteristic bands at 910 and 1710 cm^{-1}, of which the peak

at 1710 cm^{-1} did not overlay with the characteristic peaks of PMDAPRO (at 1642 cm^{-1}) and SDDPCC (at 1684 cm^{-1}) (as shown by the dotted lines in Figure S2-I, IV).

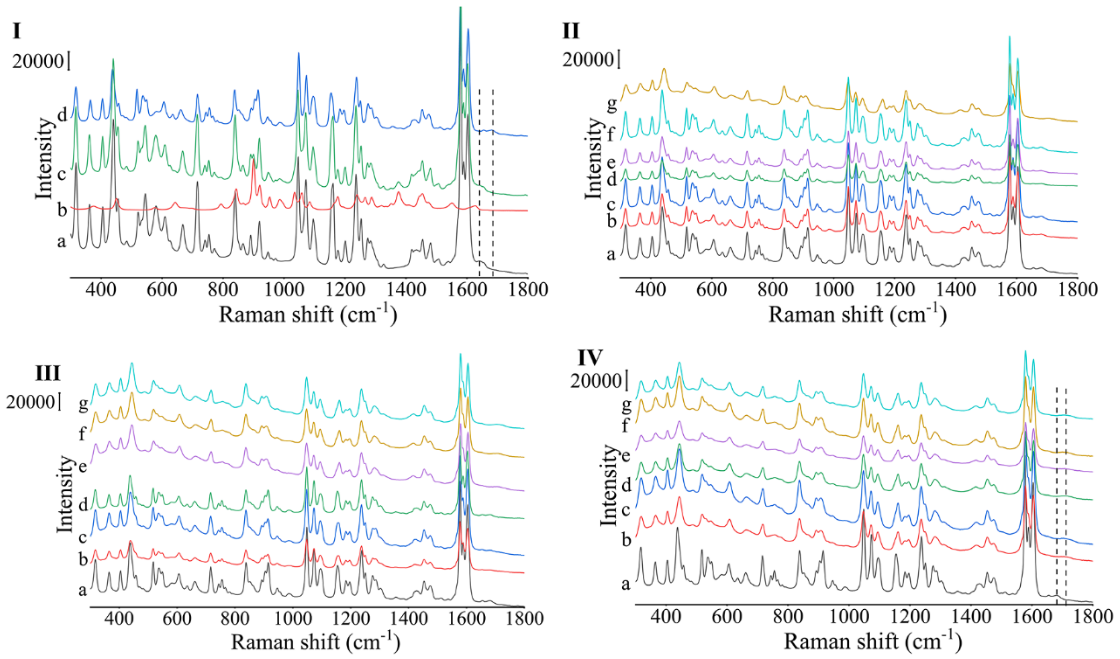

Figure S2. Raman spectra in fingerprint region collected by PhAT probe of (I): (a) DA form II, (b) PROAH, (c) PMDAPRO, (d) SDDPCC. (II): (a) SDDPCC, (b) SDPVP1, (c) SDPVPVA1, (d) SDHPMC1, (e) SDHAS1, (f) SDEC1, (g) SDEUD1. (III): (a) SDDPCC, (b) SDPVP3, (c) SDPVPVA3, (d) SDHPMC3, (e) SDHAS3, (f) SDEC3, (g) SDEUD3. (IV): (a) SDDPCC, (b) SDPVP5, (c) SDPVPVA5, (d) SDHPMC5, (e) SDHAS5, (f) SDEC5, (g) SDEUD5

Table S3. The major Raman band shifts from DA form II, PROAH, and PMDAPRO, to DPCC in the fingerprint region.

Raman bond stretching frequency (1/cm)	*Intensity*
1654, 1642 (DA form II) → 1654, 1684 (DPCC)	Small → Very small
1177 (DA form II, PROAH) → 1184 (DPCC)	Medium small → Medium
919 (DA form II) → 915 (DPCC)	Medium → Medium
900 (PROAH) → 905 (DPCC)	Medium → Medium
772 (DA form II) → 766 (DPCC)	Small → Small
579 (DA form II) → 581 (DPCC)	Medium high → Small
533 (DA form II) → 537 (DPCC)	Medium → Medium high

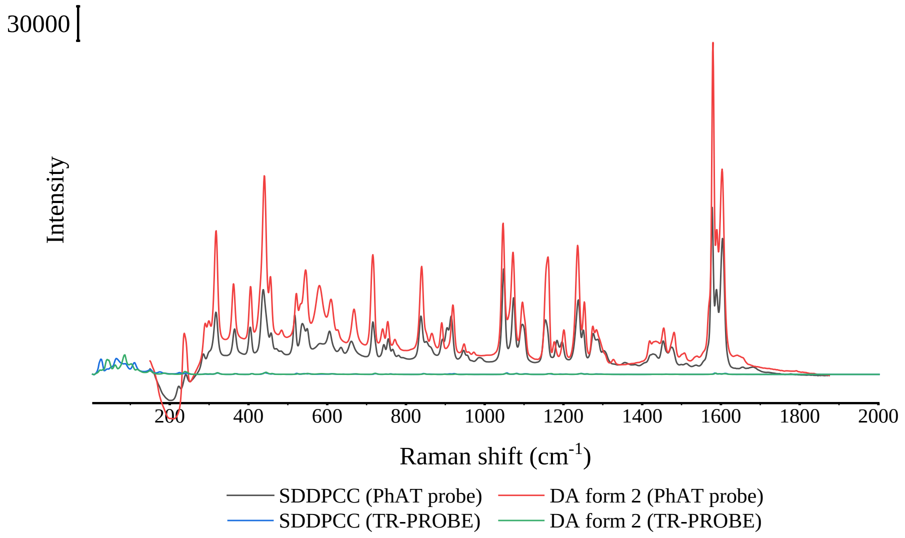

Figure S3. Raman spectra collected using the PhAT probe of SDDPCC (black spectrum) and DA form II (red spectrum), and Raman spectra collected using the THz-PROBE of SDDPCC (blue spectrum) and DA form II (green spectrum)

1.4 Raman analysis in low-frequency region

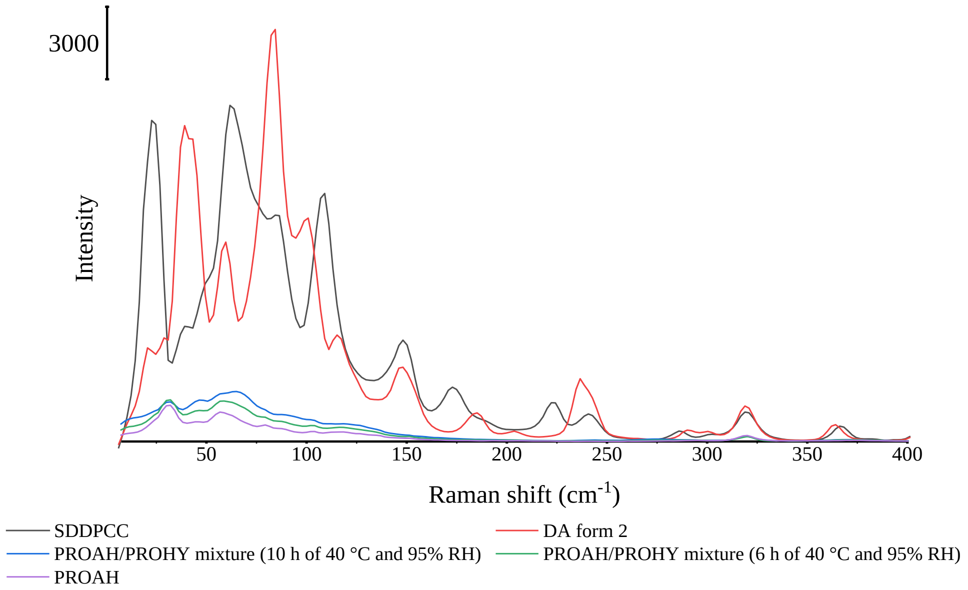

Figure S4, Raman spectra collected using the THz-PROBE of SDDPCC (black spectrum), DA form II (red spectrum), PROAH/PROHY mixture after 10 h at 40 °C and 95% RH (blue spectrum), PROAH/PROHY mixture after 6 h at 40 °C and 95% RH (blue spectrum), and PROAH (purple spectrum)

Supporting information

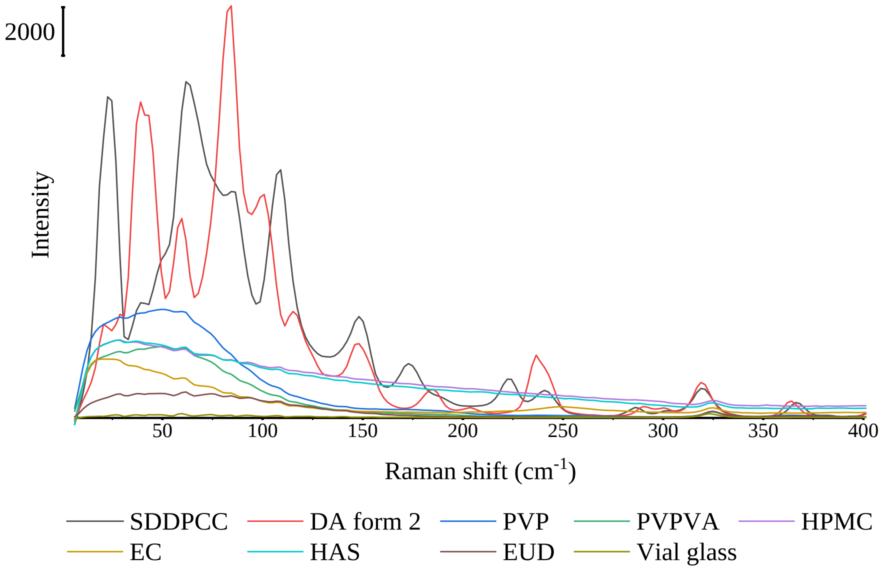

Figure S5, Raman spectra collected using the THz-PROBE of SDDPCC (black spectrum), DA form II (red spectrum), PVP (blue spectrum), PVPVA (green spectrum), HPMC (purple spectrum), EC (grey spectrum), HAS (cyan spectrum), EUD (brown spectrum), and the glass of the vial (army green spectrum).

Table S4, Correlation of most-intense experimental and ss-DFT simulated Raman-active vibrational frequencies (cm^{-1}) for DPCC, along with descriptions of the vibrational characters. Crystallographic axes are provided as needed.

**Exp. Freq. (cm^{-1})**	**ss-DFT (cm^{-1})**	**Vibrational Description**
19.1	17.83	PRO rotation about *b*<br>DA rotation about *a*
25.0	23.94	PRO translation along *a*<br>DA rotation about *b*
54.6	56.93	PRO rotation about *b*<br>DA rotation about *b*
60.2	61.89	PRO ring torsion at C=O
63.2	64.95	PRO translation along *c*<br>DA rotation about *b*
71.7	69.81<br>75.39	DA torsion of carboxylic acid chain (69.81)<br>DA torsion of rings at amine linkage (75.39)<br>PRO rotation about *b* (75.39)
81.3	81.14<br>83.95	PRO rotation about *a* (81.14)<br>DA ring-ring torsion - phase related (81.14)<br>DA ring-ring torsion - phase related (83.95)
90.4	88.02<br>93.46	PRO translation in *ab*-plane (88.02)<br>DA ring-ring torsion (93.46)<br>PRO out-of-plane bending (93.46)
113.4	109.53<br>114.72	DA ring bending at amine linkage (109.53)<br>PRO ring twisting (114.72)
151.1	146.99<br>154.05	DA phenylacetic acid ring torsion (146.99)<br>DA intra-chain torsion (154.05)
175.0	175.66	DA ring-chain torsion at amine linkage

1.5 QPA model in low-frequency Raman region

There are two concerns with applying the calibration curve to estimate how fast the cocrystal dissociates.

Firstly, DA has three polymorphs, and PRO has two solid state forms, PROAH and PROHY (anhydrous and monohydrate). Although DPCC could dissociate to any of the above-mentioned crystalline forms, the calibration curve was only based on DA form II

and DPCC, so the presence of other crystalline polymorphs could affect the calculations. PROAH and PROHY, on the other hand, appeared to have little effect on the spectra and should not affect the estimation. The PM of DA form II and PROAH at a stoichiometric molar ratio of 1:1 presented the same spectrum as DA form II (Figure 3), indicating that PROAH itself would not affect the spectra or the calculation. Unfortunately, we were unable to produce pure PROHY by following a previously reported method,[1] but we succeeded in producing a mixture of PROHY and PROAH (Figure S7) by exposing PROAH to 40 °C and 95% RH for 10 hours. With an increased proportion of PROHY in the mixture, the intensity of the spectrum was still weak at wavenumbers lower than 40 cm^{-1}, like PROAH (Figure S4), and the spectra of PROAH and the mixture of PROAH and PROHY were alike, so the possible existence of PROHY was not a concern for the estimation. With regard to the possible existence of DA form I and form 3, we utilized PXRD to monitor the crystalline phases in the solids, and for any solid that presented any Bragg peaks representing a phase that is not DA form II, DPCC, PROAH, or PROHY an estimation of cocrystal% was not made.

Secondly, the solid dispersions also contain polymer, which might affect the estimation. However, as there was only a maximum of 5% cocrystal weight of the polymer in solid dispersions and the polymer did not present a high Raman scatter (Figure S5) below 100 cm^{-1}, the polymer contribution to the spectra of solid dispersions should be small and can be ignored for the estimation of cocrystal%. The glass of the vials used for the stability study did not present any peaks below 100 cm^{-1} (Figure S5), indicating that any glass signal that might be taken by the laser would not affect the spectra and the estimation of cocrystal% by the calibration curve.

I

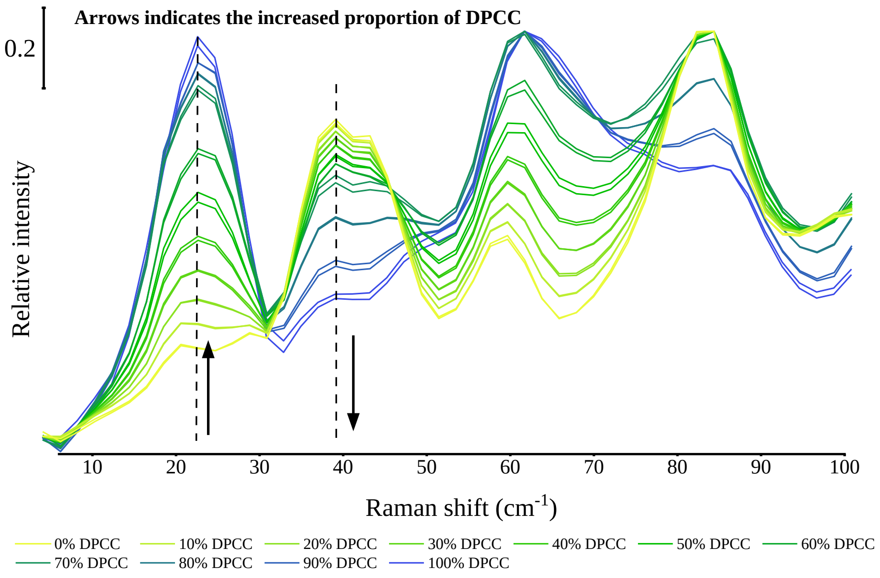

II

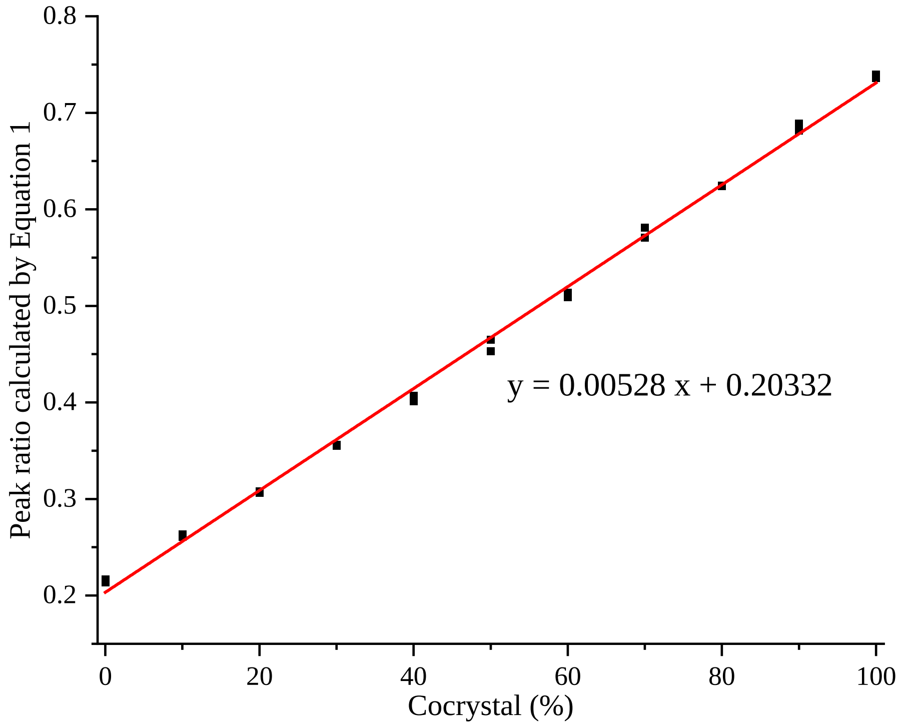

Figure S6, (I) Raman spectra below 400 cm^{-1} collected using the THz-PROBE of the PMs with different proportions of DPCC and DA form II. (II) the calibration curve was built using the value calculated by Equation (Eq. 1) (y-axis) and the proportion of DPCC in the PM (x-axis).

1.6 PXRD analysis

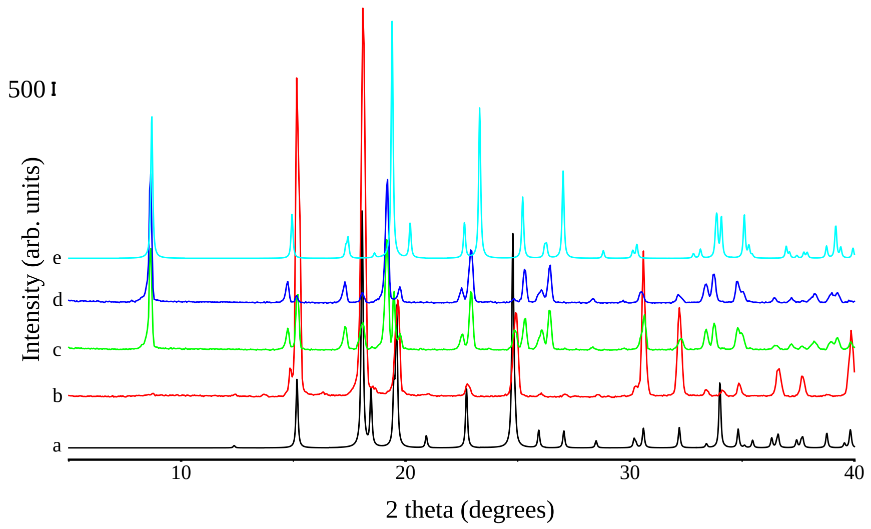

Figure S7. PXRD patterns of (b) PROAH, (c) PROAH/PROHY mixture after PROAH being held at 40 °C and 95% RH for 6 h, (d) PROAH/PROHY mixture after PROAH being held at 40 °C and 95% RH for 10 h. Simulated (from single crystal x-ray) PXRD patterns of (a) PROAH, (e) PROHY

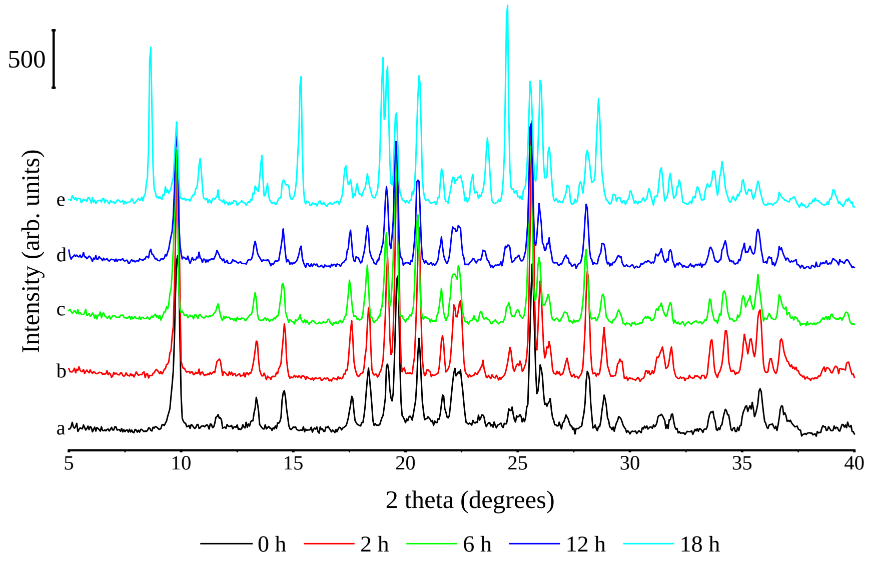

Figure S8. PXRD patterns of SDDPCC exposed to 40 °C and 95% RH for (a) 0 h (black pattern), (b) 2 h (red pattern), (c) 6 h (green pattern), (d) 12 h (blue pattern), (e) 18 h (cyan pattern)

Supporting information

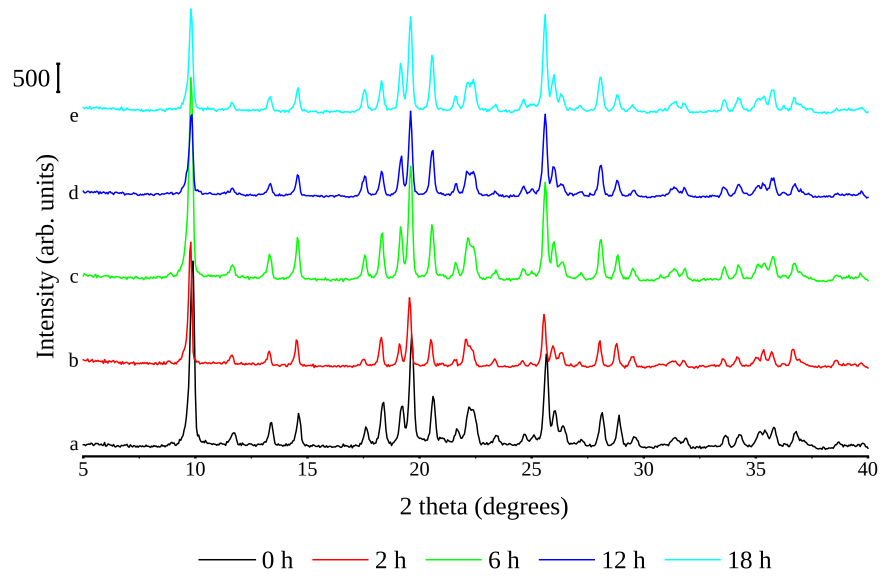

Figure S9. PXRD patterns of SDPVP1 exposed to 40 °C and 95% RH. (a) 0 h (black pattern), (b) 2 h (red pattern), (c) 6 h (green pattern), (d) 12 h (blue pattern), (e) 18 h (cyan pattern)

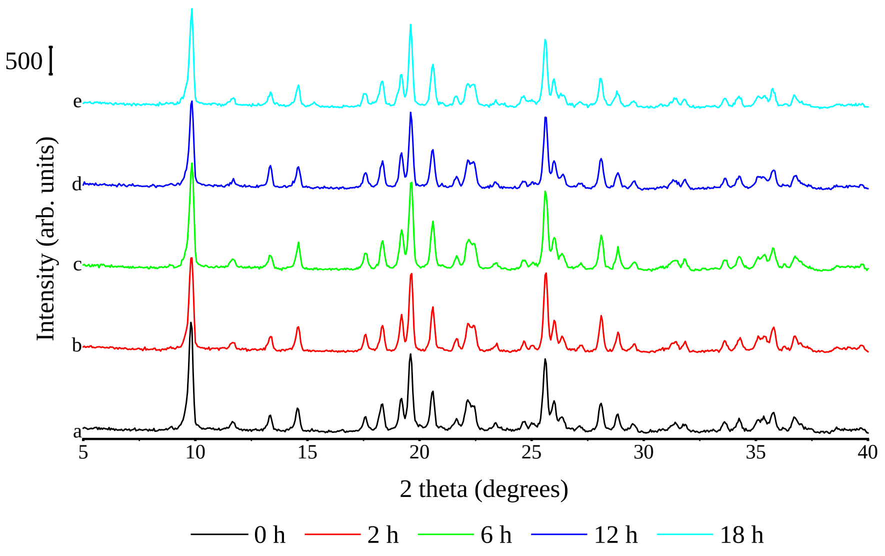

Figure S10. PXRD patterns of SDPVPVA1 exposed to 40 °C and 95% RH. (a) 0 h (black pattern), (b) 2 h (red pattern), (c) 6 h (green pattern), (d) 12 h (blue pattern), (e) 18 h (cyan pattern)

Supporting information

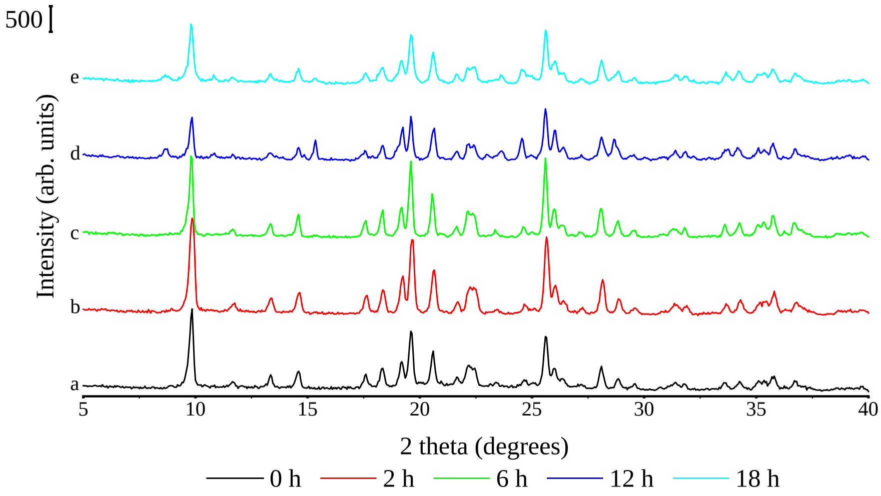

Figure S11. PXRD patterns of SDHPMC1 exposed to 40 °C and 95% RH. (a) 0 h (black pattern), (b) 2 h (red pattern), (c) 6 h (green pattern), (d) 12 h (blue pattern), (e) 18 h (cyan pattern)

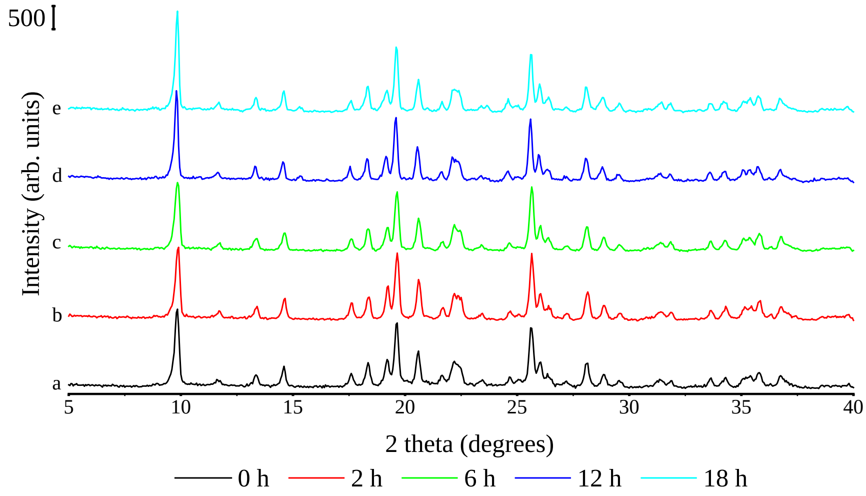

Figure S12. PXRD patterns of SDHAS1 exposed to 40 °C and 95% RH. (a) 0 h (black pattern), (b) 2 h (red pattern), (c) 6 h (green pattern), (d) 12 h (blue pattern), (e) 18 h (cyan pattern)

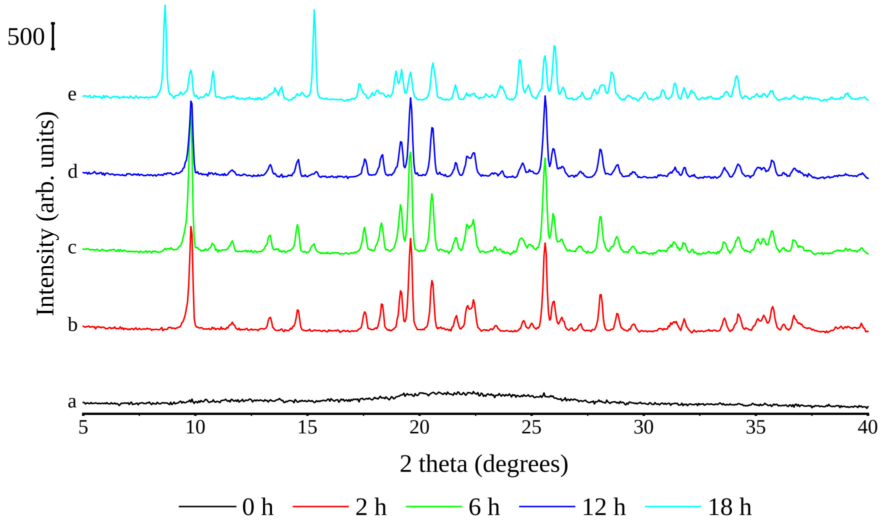

Figure S13. PXRD patterns of SDEC1 exposed to 40 °C and 95% RH. (a) 0 h (black pattern), (b) 2 h (red pattern), (c) 6 h (green pattern), (d) 12 h (blue pattern), (e) 18 h (cyan pattern)

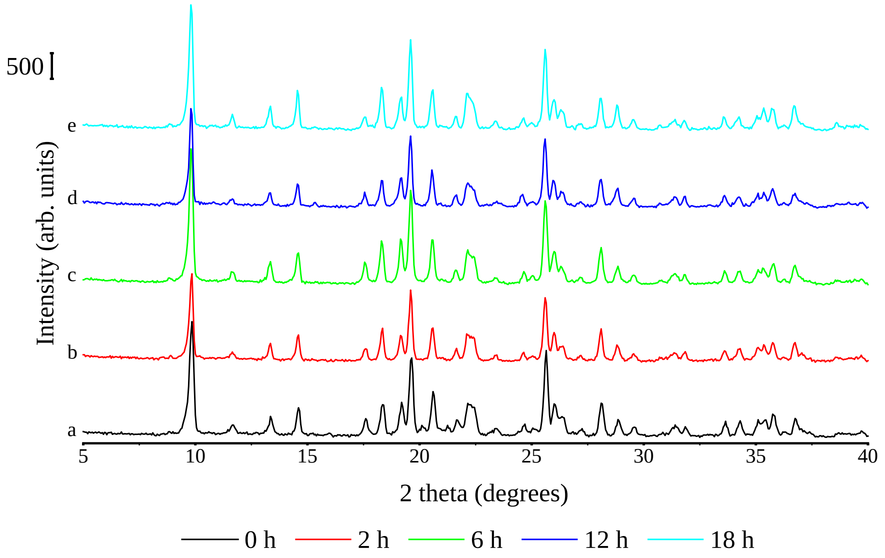

Figure S14. PXRD patterns of SDEUD1 exposed to 40 °C and 95% RH. (a) 0 h (black pattern), (b) 2 h (red pattern), (c) 6 h (green pattern), (d) 12 h (blue pattern), (e) 18 h (cyan pattern)

Supporting information

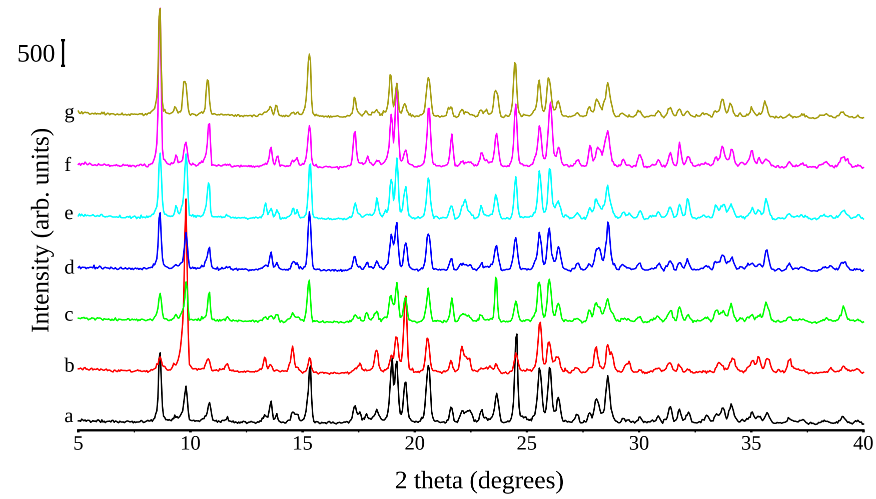

Figure S15. PXRD patterns of SDDPCC and PMs (Table 2) exposed to 40 °C and 95% RH for 18 h. (a) SDDPCC, (b) PMPVP1, (c) PMPVPVA1, (d) PMHPMC1, (e) PMHAS1, (f) PMEC1, (g) PMEUD1

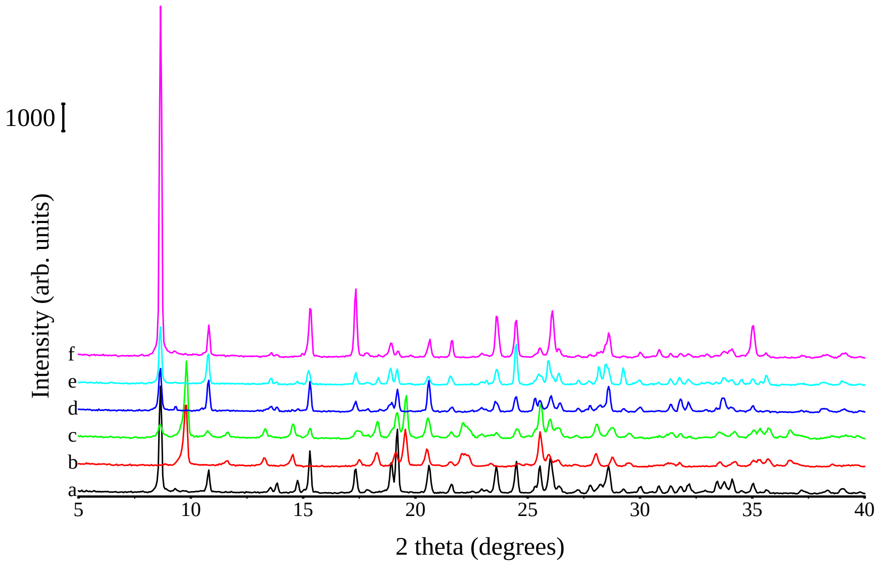

Figure S16. PXRD patterns of SDDPCC and CSDs (Table 3) exposed to 40 °C and 95% RH for 7 days. (a) SDDPCC, (b) SDPVP1, (c) SDPVPVA1, (d) SDHPMC1, (e) SDHAS1, (f) SDEUD1

1.7 FTIR analysis

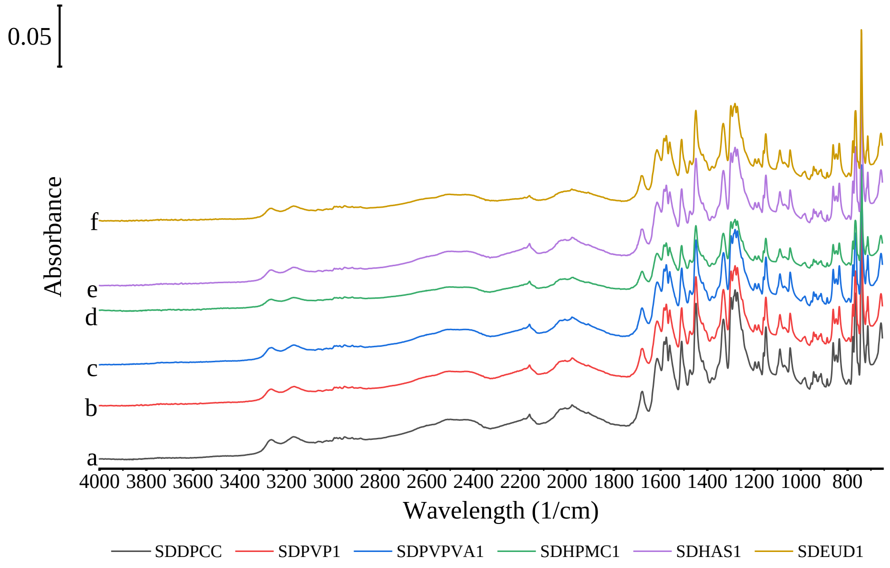

Figure S17. FTIR spectra of (a) SDDPCC, (b) SDPVP1, (c) SDPVPVA1, (d) SDHPMC1, (e) SDHAS1, (f) SDEUD1

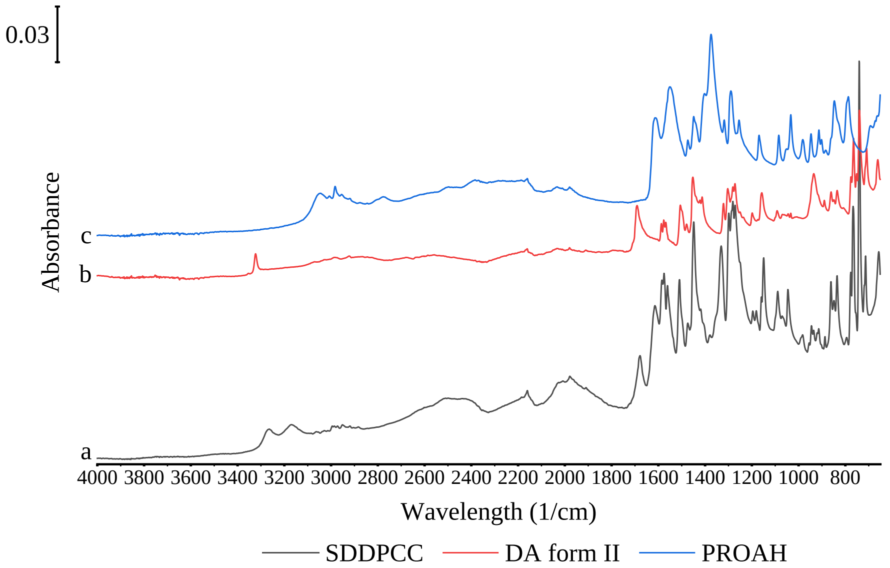

Figure S18. FTIR spectra of (a) SDDPCC, (b) DA form II, (c) PROAH

1.8 Particle size analysis

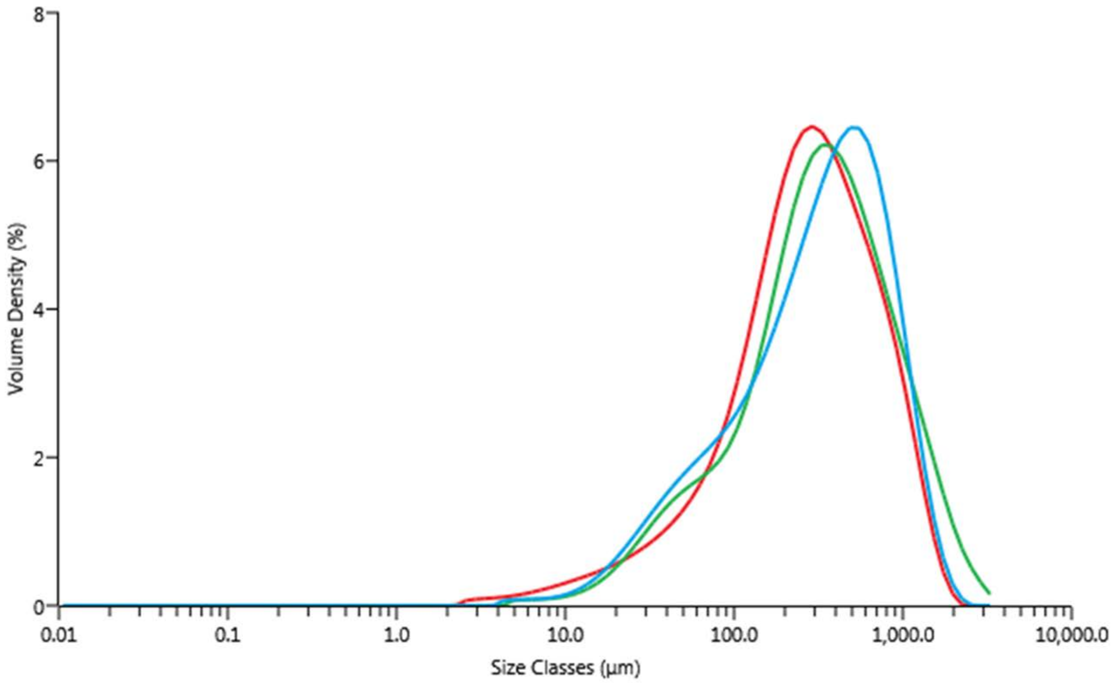

Figure S19. Particle size distribution of SDDPCC.

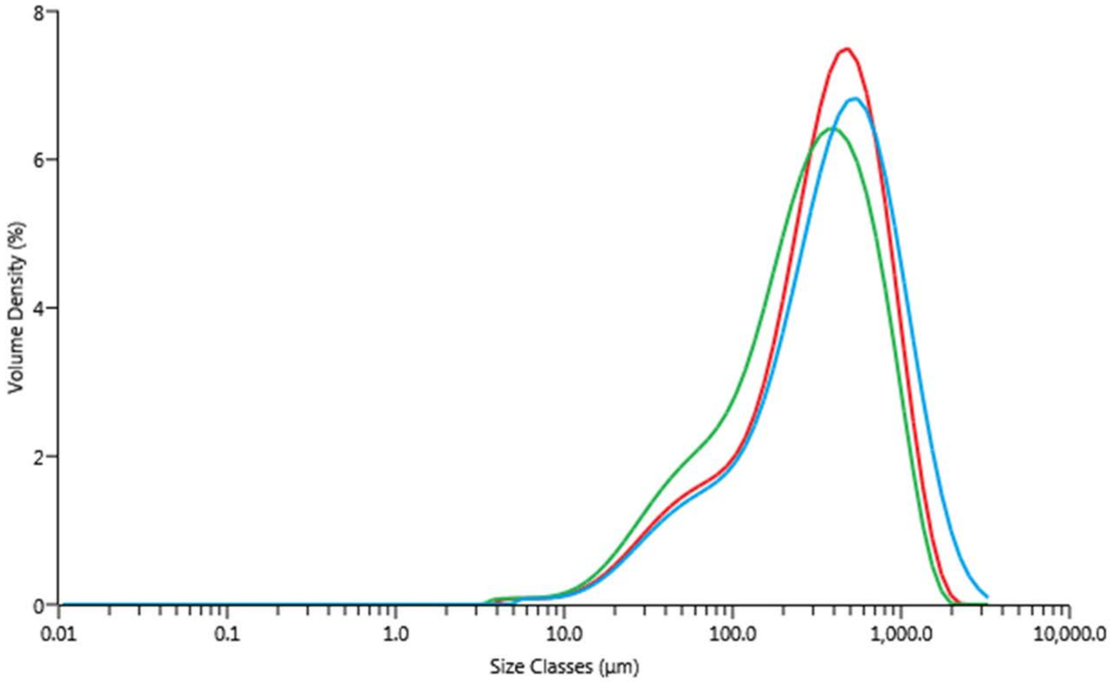

Figure S20. Particle size distribution of SDPVP1.

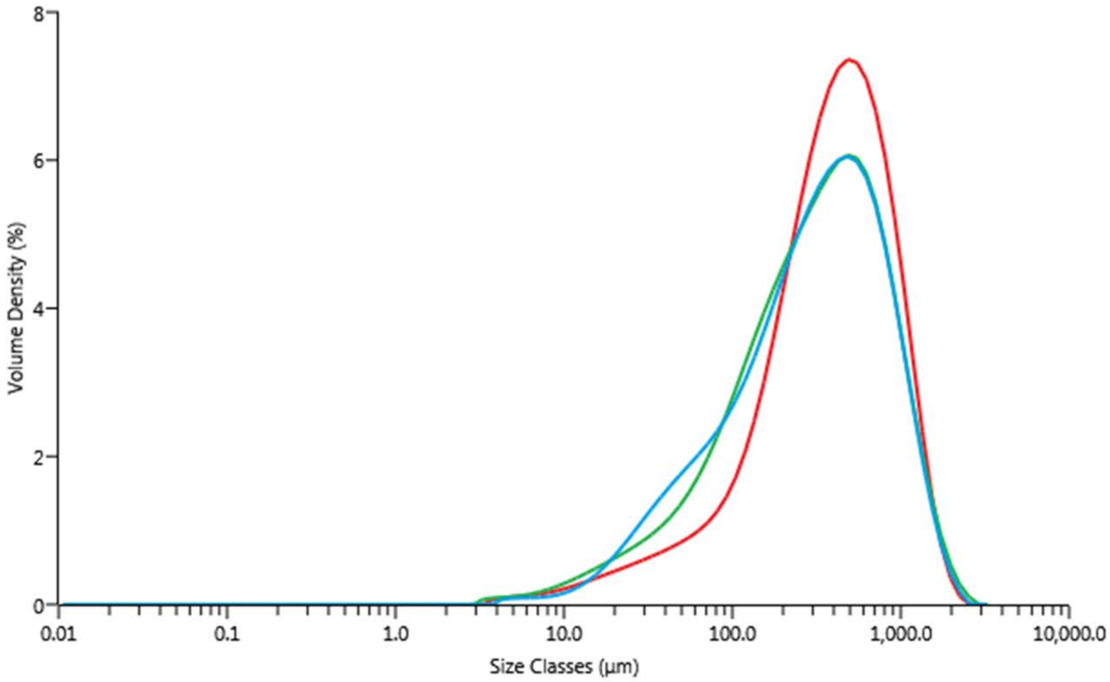

Figure S21. Particle size distribution of SDPVPVA1.

Table S5. Particle size analysis of SDDPCC, SDPVP1 and SDPVPVA1.

	d10 (μm)	*d50 (μm)*	*d90 (μm)*
SDDPCC	57.3 ± 4.0	310.4 ± 30.2	955.3 ± 123.6
SDPVP1	65.5 ± 19.1	343.4 ± 46.9	964.6 ± 28.1
SDPVPVA1	59.8 ± 9.2	350.0 ± 61.5	936.3 ± 163.5

1.9 SEM

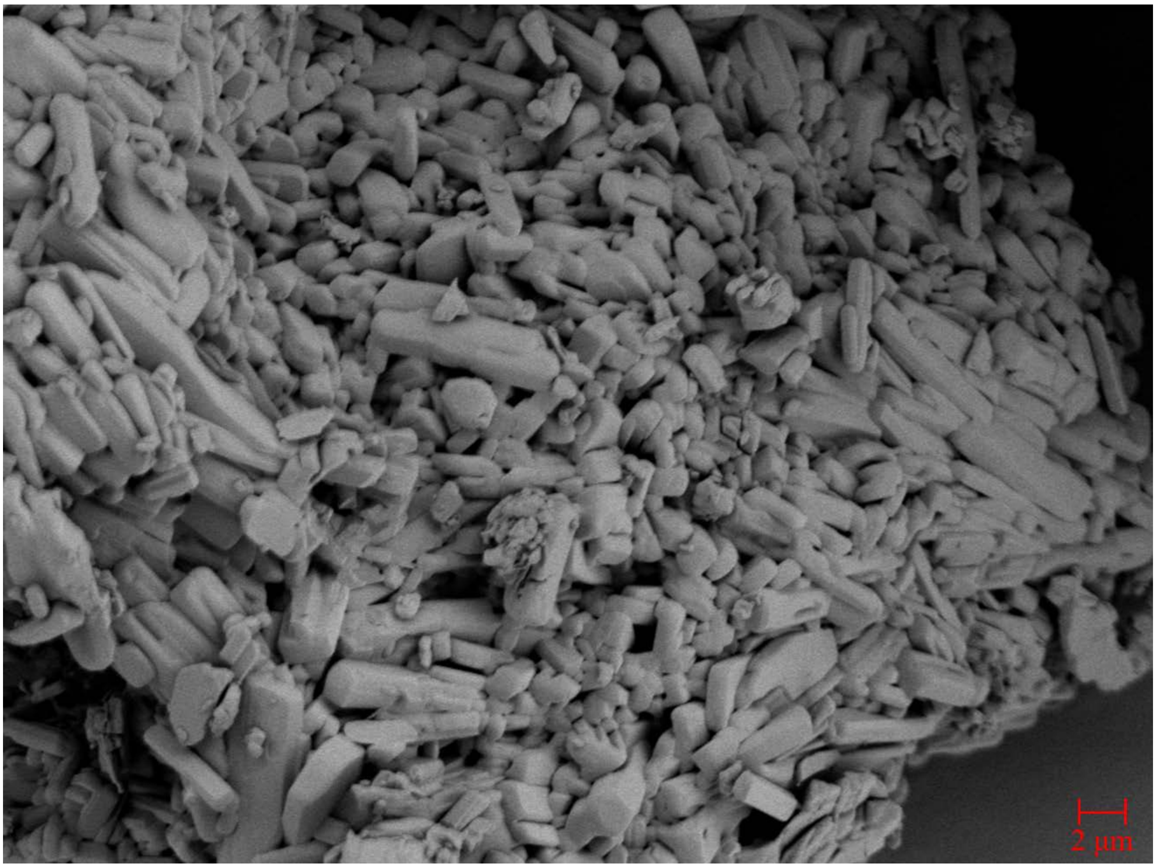

Figure S22. SEM of SDDPCC.

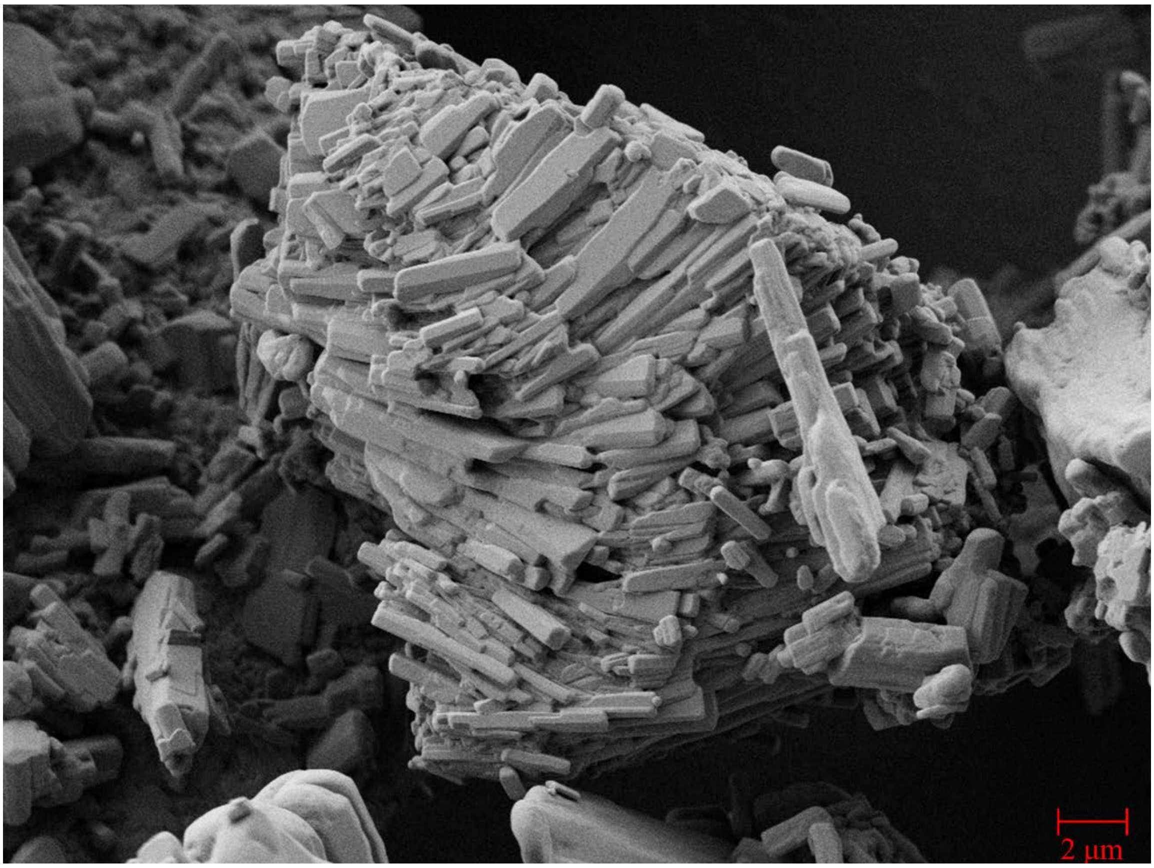

Figure S23. SEM of SDPVP1

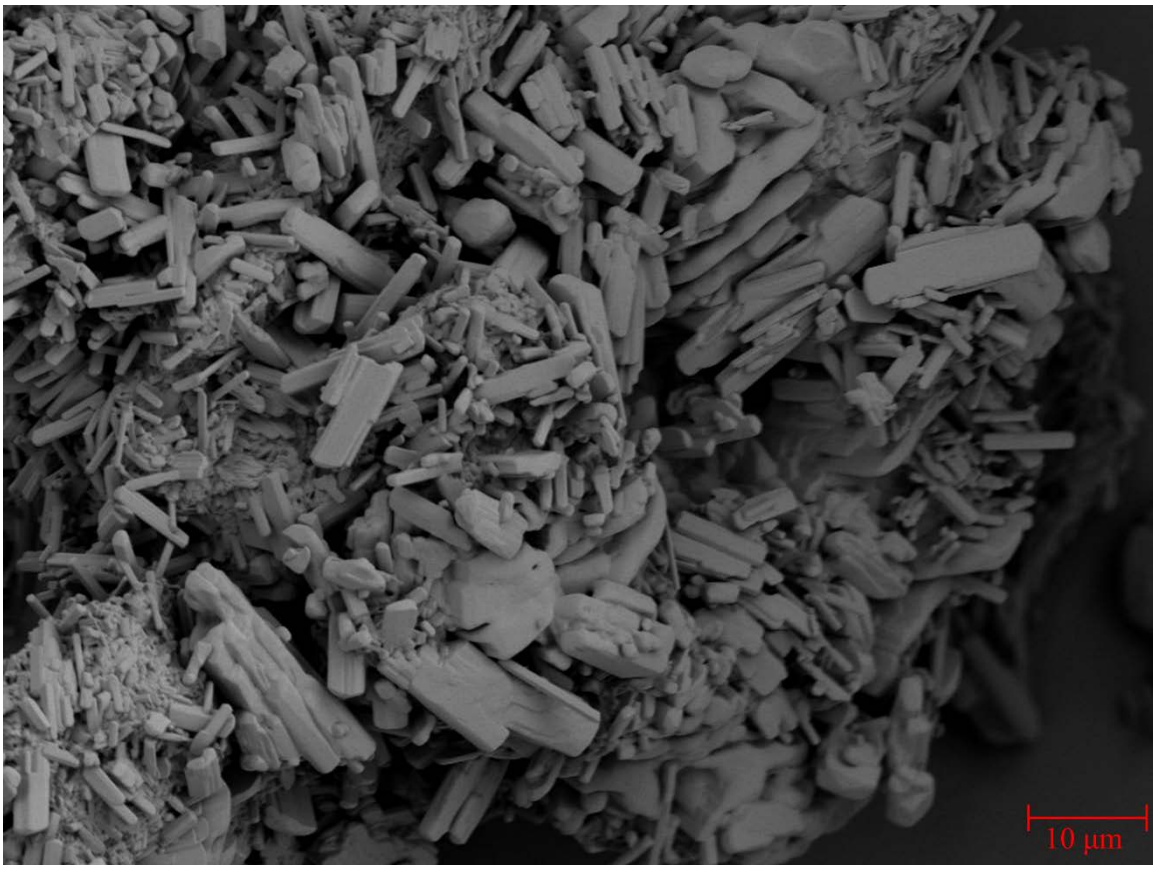

Figure S24. SEM of SDPVPVA1

1.10 Imaging of powder in bulk

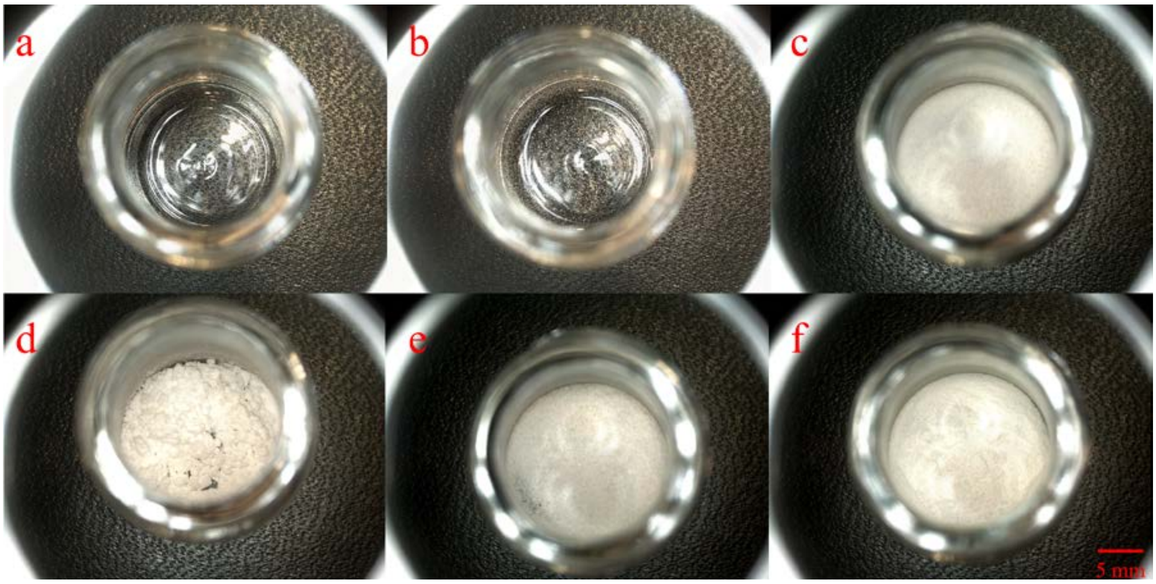

Figure S25. Imaging of 100 mg of powder after 18 h at 40 °C and 95% RH (Polymer hygroscopicity study). (a) PVP, (b) PVPVA, (c) HPMC, (d) HAS, (e) EC, (f) EUD.

1.11 Dynamic solubility in water

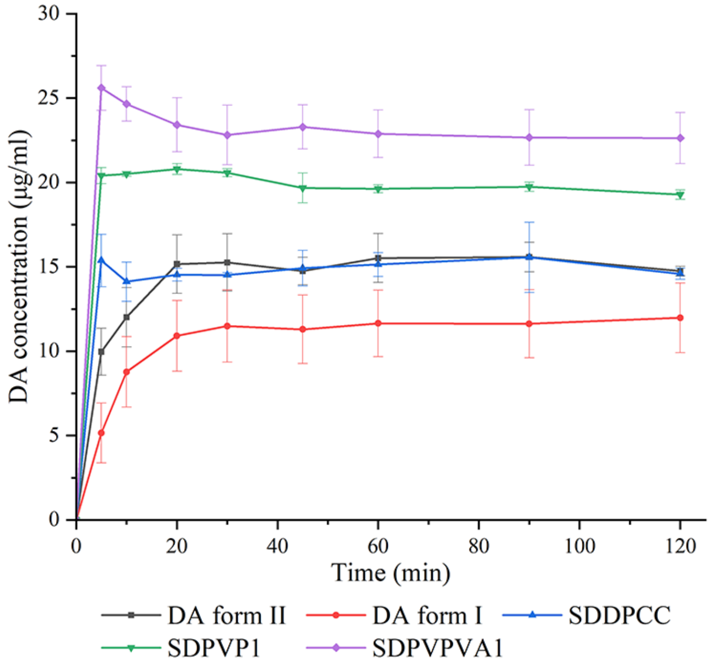

Figure S26. Dynamic solubility of DA form II (black profile), DA form I (red profile), SDDPCC (blue profile), SDPVP1 (green profile), and SDPVPVA1 (purple profile) in water

Table S6. QPA estimation of SDDPCC, SDPVP1, and SDPVPVA1 after 2 h of dynamic solubility study in water. *: Diffractograms only show one phase, so Rietveld refinement was not analyzed. N/A: not applicable.

Solid form/dispersions	*QPA by Rietveld refinement*	*Rwp*	*GOF*
DA form II	100% DA form II	N/A*	N/A*
DA form I	100% DA form I	N/A*	N/A*
SDDPCC	7.1% DA form I + 92.9% DA form II	12.81	5.32
SDPVP1	86.7% DA form I + 13.3% DA form II	10.54	2.54
SDPVPVA1	83.3% DA form I + 16.7% DA form II	11.90	2.91

1.12 pH measurement

Table S7, the pH of the medium of 2 h of dynamic solubility study. N/A: not applicable

Solid form/dispersions (dissolved polymer)	*pH after dynamic solubility study in pH 6.8 phosphate buffer*	*pH after dynamic solubility study in water*
DA form II	6.8	5.8
DA form I	6.8	6.1
SDDPCC	6.8	5.4
PMDAPRO	6.8	N/A
SDPVP1	6.8	5.6
SDPVPVA1	6.8	5.6
PMPVP1	6.8	N/A
PMPVPVA1	6.8	N/A
PMPVP10	6.8	N/A
PMPVPVA10	6.8	N/A

1.13 References

(1) Janczak, J.; Luger, P. L-Proline Monohydrate at 100 K. *Acta Crystallogr. Sect. C* **1997**, *53* (12), 1954–1956. https://doi.org/10.1107/S0108270197011487.
